# A Revised Model of Anatomically Modern Human Expansions Out of Africa through a Machine Learning Approximate Bayesian Computation Approach

**DOI:** 10.3390/genes11121510

**Published:** 2020-12-16

**Authors:** Maria Teresa Vizzari, Andrea Benazzo, Guido Barbujani, Silvia Ghirotto

**Affiliations:** Department of Life Sciences and Biotechnology, University of Ferrara, 44121 Ferrara, Italy; mariateresa.vizzari@unife.it (M.T.V.); andrea.benazzo@unife.it (A.B.); guido.barbujani@unife.it (G.B.)

**Keywords:** approximate Bayesian computation, demographic history, human evolution, migration, machine learning, random forest, whole-genome data

## Abstract

There is a wide consensus in considering Africa as the birthplace of anatomically modern humans (AMH), but the dispersal pattern and the main routes followed by our ancestors to colonize the world are still matters of debate. It is still an open question whether AMH left Africa through a single process, dispersing almost simultaneously over Asia and Europe, or in two main waves, first through the Arab Peninsula into southern Asia and Australo-Melanesia, and later through a northern route crossing the Levant. The development of new methodologies for inferring population history and the availability of worldwide high-coverage whole-genome sequences did not resolve this debate. In this work, we test the two main out-of-Africa hypotheses through an Approximate Bayesian Computation approach, based on the Random-Forest algorithm. We evaluated the ability of the method to discriminate between the alternative models of AMH out-of-Africa, using simulated data. Once assessed that the models are distinguishable, we compared simulated data with real genomic variation, from modern and archaic populations. This analysis showed that a model of multiple dispersals is four-fold as likely as the alternative single-dispersal model. According to our estimates, the two dispersal processes may be placed, respectively, around 74,000 and around 46,000 years ago.

## 1. Introduction

Levels and patterns of genome diversity reflect past demographic processes, and a crucial turning point in our demographic history is the expansion of anatomically modern humans (AMH) from Africa. Some aspects of this process seem rather well established. First, what is often called the ancestral African population should not be regarded as a single, biologically homogeneous unit, but as a structured population hosting regional diversity [1]. Second, the AMH expansion was accompanied by the disappearance of preexisting archaic human forms [2,3] Third, a variable component of the genomes of most present populations—always small, seldom zero—comes from anatomically archaic ancestors [4].

Conversely, there is disagreement over other aspects of the AMH expansion out of Africa, such as the number of major dispersal events, their timing, and the geographical routes followed by migrating people. Groups of AMH may have left Africa more than 100,000 years ago [5], but genetic evidence suggests that such early phenomena were not successful and did not lead to the establishment of permanent non-African populations. One expansion left traces in modern genomes; it took place between 60,000 and 50,000 years ago, along a Northern route in the Nile valley and across the Near East (see e.g., [6,7,8]). However, based on cranial morphology, Lahr and Foley [9] proposed an additional, earlier migration through a Southern route, from the Horn of Africa into the Arab peninsula, Southern Asia, and Australo-Melanesia. We shall refer to these alternative models as Single Dispersal (SD) and Multiple Dispersal (MD) hypotheses. The MD hypothesis found support in several studies, and notably in a comparison of cranial and DNA diversity data [10] but broader genomic analyses gave contradictory results. Tassi and colleagues [11] and, to a lesser extent, Pagani et al. [12] described patterns consistent with two dispersal processes, the first one overlapping in time with the proposed early Southern exit from Africa [11]. On the other hand, two studies of different genomic datasets concluded that there is little [4] or no evidence [13] for such an early dispersal process, and hence that AMH either left Africa in a single major migrational wave, or perhaps in several waves, but then only one of them contributed to the ancestry of modern populations.

Malaspinas et al. [13] conclusion in favor of SD was not really based on an explicit comparison between models. In their paper, indeed, they considered an MD model in which East Asians and Europeans have a more recent common ancestor than Aboriginal Australians and East Asians. and they estimated the models’ parameters. The evidence supporting the SD model came from the overlapping estimation for the divergence times of the ancestors of Aboriginal Australians and Eurasians.

This non-straightforward procedure was due to an implicit limitation of the composite likelihood method they applied, in which model selection may be performed through likelihood ratio tests (LRT) or by the Akaike Information Criterion (AIC; [14,15]). LRT and AIC can only be used to understand which modifications significantly improve the model, without explicit model testing and a direct attribution of probabilities to each tested scenario.

To understand which model, SD or MD, better accounts for the current levels of genome diversity, in this study we formally compare them by a recently developed Approximate Bayesian Computation framework, based on the study of the observed Frequency Distributions of four categories of Segregating Sites for pair of populations (FDSS) [16]. ABC is a powerful and flexible framework, based on computer simulations, to perform model selection and estimate models’ parameters. In its original formulation [17,18] the ABC algorithm suffered from two main issues, related to the simulation effort and to the number of summary statistics used to summarize the data. These issues limited the possibility to use ABC for the analysis of complex demographic histories and/or large datasets. In 2015, the introduction of a paradigm shift in the ABC model selection procedure based on a Machine Learning approach called Random Forest (ABC-RF, [19]), allowed to overcome the above-cited limitations and paved the ground for the application of ABC to the study of complex models through the analysis of complete genomes. Under ABC-RF, the model selection procedure is rephrased as a classification problem. At first, the classifier is constructed from simulations from the prior distribution via a machine learning RF algorithm. Once the classifier is constructed and applied to the observed data, the posterior probability of the resulting model can be approximated through another RF that regresses the selection error over the statistics used to summarize the data. The number of simulations necessary to obtain reliable estimates passed from a few million to a few thousand; the informative statistics are systematically extracted from the pool used to summarize the data. In 2018, a similar approach, based on a machine-learning tool of regression RF, has been developed for parameter estimation [20]. In [16] we showed that the ABC-RF algorithm, combined with the inferential power provided by the FDSS, can be satisfactorily exploited to estimated past population dynamics even in case of complex demographic histories, thus making the approach particularly suitable to the analysis of SD and MD models.

Under both SD and MD models, the structure of the past populations is the same, but the tree topologies differ in that they assume, respectively, one ancestral population for the SD model, and two ancestral populations leaving Africa at different times for the MD model. As the Australo-Melanesian represent the population that might carry the signal of the first wave of migrations out of the African continent and also, to make sure that the different results obtained by [12,13] were not due to differences in the Australo-Melanesian samples available, we repeated our analyses considering genomes coming from both studies, obtaining results that seem consistent and informative.

## 2. Materials and Methods

### 2.1. The FDSS

We summarized the data through the FDSS, i.e., the frequency distributions of the four mutually exclusive categories of segregating sites for pair of populations (i.e., private polymorphisms in either population, shared polymorphisms, and fixed differences [21]). This statistic proved to be powerful for reconstructing even a complex series of demographic processes [16]. The FDSS is calculated considering each genome analyzed as subdivided into a certain number of independent fragments of a certain length, and for each fragment, the number of sites belonging to each of the four above-mentioned categories is counted. The final vector of summary statistics is thus composed by the truncated frequency distribution of fragments having from 0 to n segregating sites in each category, for each pair of populations considered. We fixed the maximum number of segregating sites in a locus of a certain length to 100, and hence the last category contains all the observations higher than 100.

We calculated the FDSS using a python script (available on Github https://github.com/anbena/ABC-FDSS) [16]. The ABC-RF model selection estimates have been obtained using the function *abcrf* from the package *abcrf* and employing a forest of 500 classification trees, a number suggested providing the best trade-off between computational efficiency and statistical precision [19]. Before proceeding with the model selection procedure, we computed the confusion matrices and evaluated the out-of-bag classification error (CE) and the proportion of True Positives (1-CE), which are representative of the power of the whole inferential procedure. The ABC-RF parameters estimation on the most supported models have been performed through the function *regAbcrf* from the package *abcrf* and employing a forest of 500 regression trees. An outline of our entire workflow is reported in Figure S1.

### 2.2. Simulated Models of Anatomically Modern Humans Expansion Out of Africa

**Figure 1:**
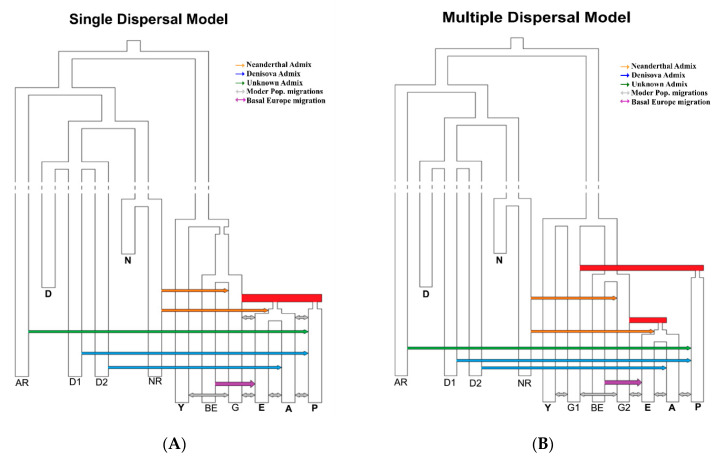
Demographic models compared: Single Dispersal (**A**) and Multiple Dispersals (**B**). AR: unknown archaic population; D-D1-D2: Denisovan groups; N-NR: Neandertal and Neandertal related groups; Y: African population; G1-G2: ghost populations; BE: Basal Europe population; E: European population; A: Asian population; P: Australo-Melanesian population.

We tested two alternative models of expansion of anatomically modern humans out of the African continent (Figure 1), both sharing the same structure for the archaic groups, but differing for the relationships among modern populations. To design the models, we followed the parametrization proposed by [13], with some modifications detailed below. The first model (SD) indeed accounts for a single dispersal from Africa giving rise to both modern Eurasians and Australo-Melanesians, the second model (MD) accounts for two different waves of migrations, from two different African source populations, giving rise, first, to the modern Australo-Melanesians and, later to the modern Eurasians. The archaic groups consist of three Denisovan populations, two Neanderthal populations, and an unknown archaic population ancestral to both Neandertals and Denisovans. We explicitly considered admixture pulses from archaic to modern populations: a pulse from the archaic unknown population to Australo-Melanesians (as reported in [22]), two pulses from two different Denisovan populations to Asians and Australo-Melanesians [23,24], two pulses from the same Neandertal population to modern humans just after the separation between African and non-African populations, and to the ancestor of all Eurasians [25,26,27]. Both models account for the presence of a Basal European population, as described in [28,29,30]. This (so far, unknown) population contributed genes to modern Europeans, possibly diluting the contribution of archaic Neandertal variants in European genomes. The SD and MD models have 45 and 50 free parameters (i.e., parameters whose values are defined by prior distributions), respectively. The prior distributions associated with these parameters were set following what was proposed in the recent literature by [13,23,30], and are reported in Tables S1 and S2. We considered a generation time of 29 years, and we fixed the mutation rate at 1.25 × 10^−8^ bp/generation [31] and the intra-locus recombination rate at 1.12 × 10^−8^, all values as in [13].

We performed 20,000, 50,000, and 100,000 simulations for each model with *ms* [32], to evaluate the Prior Error Rate and identify the optimum number of simulations to use. At each iteration, we sampled six diploid genomes, one Neandertal, one Denisova, one African, one European, one Asian, and one Papuan. The FDSS was calculated from 10,000 independent genomic fragments of 500 bp length.

### 2.3. Observed Genomic Data

We analyzed the high-coverage genomes of Denisova [33] and Neandertal [26], together with worldwide modern human samples from [12]. All the individuals were mapped against the human reference genome *hg19* build 37. To calculate the observed *FDSS* we only considered autosomal regions outside known and predicted genes ± 10,000 bp and outside CpG islands and repeated regions (as defined on the UCSC platform, [34]). We extracted 10,000 independent fragments of 500 bp length, separated by at least 10,000 bps in genomic regions that passed a set of minimal quality filters used for the analysis of the ancient genomes (*map35_50%*; [26,33]). We also included in the analysis of the 25 Papuan individuals published by [13]. For these individuals, we downloaded the alignments in CRAM format from https://www.ebi.ac.uk/ega/datasets/EGAD00001001634. The *mpileup* and *call* commands from *samtools-1.6* [35], were used to call all variants within the 10,000 neutral genomic fragments, using the --consensus-caller flag, without considering indels. We then filtered the initial call set according to the filters reported in [13] using *vcflib* and *bcftools* [35]. The complete set of samples used for the comparison between SD and MD are reported in Table S3. 

In each models’ comparison, we evaluated the genomic variation of one Denisova, one Neandertal, one African (Congo-pygmies), one European (Estonians), one Asian (Vietnamese), and one Australo-Melanesian (Papuans). We decided to restrict the analysis to one high coverage diploid genome per population since previous extensive analyses showed that a single individual sampled per population has a comparable discrimination power as twenty chromosomes [16]. However, to ensure the consistency of the results, we performed several model selection procedures (a) taking into account at each run one out of six Papuans from [12] or one of 25 Papuans from [13]; (b) considering alternative individuals as representative of African, European, and Asian populations (Table S4).

### 2.4. Assessment of the Quality of the Parameters Estimated

One of the most interesting features of ABC is its high flexibility for model checking, i.e., for assessing the quality of the estimates inferred from real data. This is mainly achieved through the analysis of pseudo-observed data (pods), i.e., simulated datasets generated under known conditions. To determine whether the observed data would contain enough information to estimate parameters of the multi-dimensional model tested, we exploited 1000 pods, each generated from the most supported model (i.e., the MD model) and through a known combination of demographic parameters. Using these pods, for each parameter we calculated the following indices:

- The coefficient of determination (R^2^). R^2^ is the fraction of variance of the parameters explained by the summary statistics used to build the regression model. In the absence of an established threshold value, there is a general agreement that when R^2^ < 0.10, the summary statistics do not convey enough information about the parameter estimates [36].
- The relative bias. To calculate the relative bias, we estimated the parameters for each pod with the same approach used for the observed data. The bias depends on the sum of differences between the 1000 estimates of each parameter thus obtained and the known (true) value, and it is calculated as
  $$\frac{1}{n}\overset{n}{\underset{i=1}{\sum }}\frac{\theta_{i}-\;\theta }{\theta }$$
  where *θ_i_* is the estimator of the parameter *θ* (true value), and *n* is the number of pods used (1000 in our case). Because bias is relative, a value of 1 corresponds to a bias equal to 100% of the true value.
- The root mean square error (RMSE). To calculate the RMSE we re-estimated parameters using pods. The RMSE depends the sum of squared differences between the 1000 estimates of each parameter thus obtained and the true value and it is calculated as:
  $$\sqrt{\frac{1}{n}\overset{n}{\underset{i=1}{\sum }}{\left(\theta_{i}-\theta \right)}^{2}}$$
- The factor 2, representing the proportion of the 1000 estimated median values lying between 50% and 200% of the true value.
- The 50% and 90% coverage, defined as the proportion of times that the known value lies within the 50% and the 90% credible interval of the 1000 estimates.

## 3. Results

### 3.1. Model Selection

Table 1 and Table S5 show the results of the power check of the comparison between SD and MD. Predictably, the Prior Error rate, which indicates the global quality of the ML classifier, decreases for increasing numbers of simulations in the reference table (from 20,000 to 100,000); for this reason, we decided to use 100,000 simulations for the subsequent analyses. The proportion of True Positives, that is the proportion of times the SD or the MD model is correctly recognized by the model selection procedure, is above 70% for both SD and MD, with a mean posterior probability associated with the true demography of about 75%.

Table 2 and Table S4 show the results of the model selection. Regardless of the Papuan individual considered, and the combination of non-Australo-Melanesian tested, the model selection analyses supported the MD model as the scenario best explaining the recent evolution of anatomically modern humans out of Africa, with probabilities ranging from 78 to 84%.

### 3.2. Parameters Estimation

Once identified the MD as the most probable model, we moved to estimate its parameter values maximizing the fit between observed and simulated genomic data. To do this, we exploited the recently developed ML method, based on a regression RF approach [20]. As detailed in [20], a faithful estimation of parameters’ posterior distribution may be now achieved with a reduced number of simulations (i.e., a few thousand; we used 100,000 simulations), making it feasible to also perform an accurate assessment of the quality of the parameters estimated using pods. 

Parameters were estimated from two observed datasets (one with a Papuan individual from [13] and one with a Papuan individual from [12]), those which produced the highest value of posterior probability for the MD model in the model selection (Table 3 and Table 4). The posterior plots and the definition of the parameter’s acronyms are reported in Supplementary Materials (Figures S2–S10, Table S6). The R^2^, the bias, the RMSE, the Factor 2, and the 50–90% Coverage associated with each of these parameters are shown in Table 5. As expected for complex demography, many parameters are not well estimated, as indicated by low R^2^, high bias, and high RMSE. The parameters showing better estimation quality are the effective population sizes, in particular those associated with the ancestral population of African and non-African modern humans (nYG, R^2^= 91%), and the ancestral population of modern and archaic groups (nAM, R^2^= 99%). The divergence times appear to have been estimated reasonably well, with most of R^2^s above 10%. This is true in particular for the times of the two Out of Africa events, which also show a low bias and a high Factor2 and Coverage. On the other hand, it is evident that the data tell us very little about admixture events (their timing and admixture proportions) and migration rates. Although disappointing, this is not unexpected, and high levels of uncertainty associated with these parameters were already reported [13].

The estimates for the current African effective population size (nY) is about 15,000 (median value), in agreement with previous studies [37,38]. A lower value is estimated for the Eurasians, with an effective population size of about 7000 individuals for the Europeans (nE) and of about 11,000 individuals for the Asians (nA). A bit higher is the estimate for Australo-Melanesian population: the median value of the effective population size is indeed about 25,000 individuals (nP).

The first divergence within Africa (tdYG1), that generated the source population giving rise to the first wave of migrants has been estimated about 104,000 years ago, with a 95% confidence interval between 55,000 and 141,000 years ago (and a 50% CI between 78,000 and 125,000 years ago). The first waves of migrants left Africa (tdOA1) about 74,000 years ago (95% CI: 47,000–120,000 years ago; 50% CI: 55,000–96,000 years ago), whereas the second wave of migration (tdOA2), originated from a structure generated (tdYG2) about 100,000 years ago, left Africa about 46,000 years ago (95% CI: 40,000–59,000 years ago, 50% CI: 42,000–51,000 years ago). Europeans and Asians diverged (tdEA) about 37,000 years ago. These estimates are in agreement with a previous work that considered a less realistic model and a smaller amount of genetic data [11].

## 4. Discussion

In this paper, we explicitly compared two models of AMH evolution through an ABC–RF approach based on the analysis of modern and ancient complete genomes. The two tested demographic models consider details of our evolutionary history that have been proposed in the recent literature, such as the presence of a (so far, unsampled) Basal European population contributing to the genome of recent Europeans [30], or the two distinct pulses of admixture from two different Denisovan populations to Asians and Papuans [23]. The main difference between the two scenarios regards the dynamics of expansion from Africa of AMH. According to the SD model, all non-African populations derive from a single major migration wave; on the contrary, the MD model assumes two migration waves, distinct in time and place, the first one giving rise to modern Australo-Melanesians and the other giving rise to Eurasians. Needless to say, successive processes of gene flow and admixture have certainly complicated the apparently simple patterns generated by the initial African dispersal(s). Yet, even these admittedly simplified models are complex (defined by up to 50 parameters), and the differences between them are relatively small; therefore, one could expect that it might be difficult to tell them apart. On the contrary, the ABC-RF procedure we chose provided a good discriminatory power, with a proportion of True Positives of about 70% for both AD and MD models. This TP proportion is comparable to, or higher than, that reported in previous works where simpler (and hence less realistic) models were analyzed (see e.g., [39,40]). When the two alternative models were compared, the MD model resulted consistently four-fold more probable than the SD model, no matter which Papuan (Table 2), African, European or Asian individuals were considered (Table S4), with a posterior probability estimated around 80%. The support for the MD model is marginally higher than in [16], where a comparison between two alternative, less up-to-date, evolutionary histories of AMH favored the MD model with a probability of about 75%. These results are robust to slight changes in the MD parametrization. We indeed tested also a version of MD in which Papuans derived part of their genomes from Eurasians, modeled as a single pulse of admixture occurring after the second exit (rather than through a process of continuous gene flow), the results are reported in Table S7. Even in this version, the MD appeared more supported by data than the SD model, although it appeared slightly less likely than the previous MD model when included in the general comparison.

In this work, for the first time, we also attempted to estimate the parameters of the supported model by ABC-RF. The MD model was defined by 50 free parameters, estimated through the regression random forest algorithm [20]. We also assessed the quality of these estimates through the calculation of statistics that gave us information about the inferential power of the parameter’s estimation procedure. An assessment of the quality of the estimated parameters was prohibitive so far, due to computational limits of other inferential methods, e.g., those based on composite-likelihood [41]. With ABC-RF, instead, the same reference table (made up of just a few thousand simulations) allows one to both estimate parameters and assess their quality using a subset of the simulation as “pods”. To perform the same analysis by composite-likelihood methods, one would require about 100 thousand new simulations for each pod analyzed, which means, even considering only 100 pods, billions of simulations. This large amount of simulated data often exceeds computational constraints, in particular when complex demographies are analyzed. As a consequence, in studies of complex models, no information was provided about the reliability of parameter estimates [13,42]. The procedure we applied made it possible to compensate for this drawback, as shown in Table 5.

It would have been unrealistic to expect that all 50 parameters could be reliably estimated. The migration rates among modern populations, or the proportion and timing of admixture events, for instance, proved elusive, showing a low R^2^ and high bias and RMSE values. We knew that there is an almost infinite set of parameter combinations leading to the same patterns of genome diversity, with, for instance, old small-scale admixture events, and recent larger-scale admixture events, producing, in principle, the same consequences at the genomic level. Other parameters show better estimates. This is the case of the effective population sizes, or, to a lesser extent, of the divergence times. The African, European and Asian estimates of the effective population sizes are consistent with what reported in the literature [38,43]; the higher value estimated for the Australo-Melanesian group, here represented by the Papuans, may be surprising, but it is in agreement with the harmonic mean of the effective population sizes estimated over time by [12].

The most interesting parameters are those associated with the divergence/departure from Africa. These parameters show R^2^ above 10%, good coverage, and a factor 2 of about 100%; however, their confidence intervals are huge and their posterior distributions often seem to reflect the prior range. This means that we should still take with caution these estimates and that the ABC inferential procedure, albeit powerful, shows room for improvement. The key advantage of the ABC estimation is that the “quality assessment” procedure allows the acquisition of consciousness about the quality of the estimates; nevertheless, having this in mind, we can still discuss the estimates obtained. We dated the structure of African groups that gave rise to the source populations of the migration waves from Africa about 100,000 years ago. The bottleneck of the first exit from Africa, associated with the origin of Australo-Melanesian groups, has been estimated at about 74,000 years ago, in line with the timing inferred from paleoanthropological data (70,000 years ago, [44]). The second exit, giving rise to Eurasian populations, was placed at about 46,000 years ago. This is in agreement with previous estimates from genomic data [4,38,45] and receives further support from the relatively recent arrival of modern humans in Europe suggested by much of the archaeological evidence (40–45 thousand years ago, [46,47]). Some authors proposed an even earlier presence of AMH in Europe [48]. Be that as it may, it is also plausible that large-scale gene flow processes, documented at least twice in Europe (in the Neolithic period and Bronze Age; see [49]) may have slightly reduced diversity and hence the apparent depth of the DNA genealogies, thus producing a bias towards more recent values in the estimation of divergence times. The two migration waves from Africa considered in the MD model appear to be separated in time, with no temporal overlap considering their 50% confidence interval (55,000–96,000 for the first exit and 42,000–51,000 for the second exit), and a limited overlap considering their 95% confidence interval (47,000–120,000 for the first exit and 40,000–59,000 for the second exit).

## 5. Conclusions

In this paper we extensively tested two up-to-date models of modern human expansion Out of Africa through a machine learning ABC approach. The simulated variation has been compared with those observed in ancient and modern genomes, and our results consistently supported a Multiple Dispersal Model, in which modern Australo-Melanesians derive from an earlier migration from Africa than that giving rise to Eurasians. We also estimated the parameters of the most supported model, and we concentrated our effort in assessing the quality of the estimates produced. This procedure, albeit fundamental to ensure the reliability of the estimates, it is rarely performed, due to the limitations of available inferential methods. These limitations are currently overcame by the ABC-RF procedure coupled with the FDSS statistic, which allowed us to highlight weakness and strengths of the parameters estimated. Our results indeed support that the hypothesis of two main dispersal event from Africa, separated in time and place [10,11,12], cannot be dismissed [4,13], but the quality assessment of the parameters we estimated certainly show that needs to be further explored.

## Figures and Tables

**Table 1:** Power test for model comparison using a reference table with 100,000 simulations per model.

Prior Err. Rate	True Positive SD	True Positive MD	Post. Prob. SD	Post. Prob. MD
0.26	0.73	0.75	0.75	0.73

**Table 2:** Model Selection results using Papuan individuals from [12,13]. In the first column are reported the ID of the Papuan samples used for the model choice. The second column shows the model selected by the ABC procedure. In the third and the fourth columns are reported the votes assigned to the SD and MD models by the Random-Forest algorithm. The last column shows the posterior probabilities associated with the most supported model. The samples with the highest posterior probabilities (in bold) were selected to perform the parameter estimation of the MD model.

ID_Individual	Selected Model	Votes SD	Votes MD	Post. Prob.
EGAN00001279031	MD	94	406	0.822
EGAN00001279039	MD	86	414	0.806
EGAN00001279047	MD	111	389	0.798
EGAN00001279054	MD	128	372	0.809
**EGAN00001279032**	**MD**	**90**	**410**	**0.825**
EGAN00001279040	MD	113	387	0.784
EGAN00001279048	MD	99	401	0.805
EGAN00001279033	MD	108	392	0.791
EGAN00001279041	MD	111	389	0.797
EGAN00001279049	MD	126	374	0.789
EGAN00001279034	MD	150	350	0.797
EGAN00001279042	MD	109	391	0.791
EGAN00001279050	MD	111	389	0.797
EGAN00001279035	MD	108	392	0.799
EGAN00001279043	MD	97	403	0.802
EGAN00001279051	MD	117	383	0.786
EGAN00001279036	MD	136	364	0.778
EGAN00001279044	MD	109	391	0.784
EGAN00001279052	MD	100	400	0.815
EGAN00001279037	MD	96	404	0.800
EGAN00001279045	MD	148	352	0.787
EGAN00001279053	MD	100	400	0.796
EGAN00001279038	MD	91	409	0.811
EGAN00001279046	MD	104	396	0.781
EGAN00001279055	MD	138	362	0.787
Koinb1	MD	165	335	0.810
Koinb2	MD	129	371	0.811
Koinb3	MD	175	325	0.820
Kosip1	MD	152	348	0.818
Kosip2	MD	136	364	0.788
**Kosip3**	**MD**	**123**	**377**	**0.830**

**Table 3:** Estimated parameters for the MD model using the Papuan samples from [13]. The mean and the median estimated values are listed, as well as the 90% and the 50% credible intervals. The parameters cited in the text are reported in bold.

Parameter	Mean	Median	Variance	Q (0.05)	Q (0.95)	Q (0.25)	Q (0.75)
**nAR**	**2822**	**2793**	**5.77 × 10^4^**	**2540**	**3410**	**2666**	**2914**
**nY**	**19,077**	**14,347**	**1.72 × 10^8^**	**4204**	**44,993**	**7976**	**29117**
nG1	26,191	26,995	**2.08 × 10^8^**	3253	47,385	13,670	39,819
nG2	23,473	22,275	**1.96 × 10^8^**	1903	46,649	11,151	34,663
nBE	25,612	26,269	**2.08 × 10^8^**	2731	47,604	13,394	38,160
**nE**	**13,498**	**6616**	**2.07 × 10^8^**	**627**	**42,565**	**1616**	**23,761**
**nA**	**16,360**	**11,553**	**2.25 × 10^8^**	**773**	**44,620**	**2599**	**28,065**
**nP**	**24,268**	**24,839**	**2.34 × 10^8^**	**1535**	**47,534**	**10,756**	**37,349**
**nYG**	**23,317**	**22,292**	**3.19 × 10^7^**	**17,112**	**35,456**	**19,789**	**25,425**
**nNNR**	**2424**	**2343**	**1.22 × 10^5^**	**2057**	**3001**	**2219**	**2504**
nDDR	21,360	19,680	2.00 × 10^8^	1570	46,512	9482	32,332
nDN	17,025	12,576	1.77 × 10^8^	2789	43,117	5312	27,001
nADN	19,733	16,531	2.28 × 10^8^	2108	47,465	5770	31,455
**nAM**	**18,846**	**18,745**	**1.73 × 10^6^**	**16,780**	**21,023**	**17,911**	**19,745**
rP	0.0214	0.0146	8.36 × 10^−4^	0.0105	0.0532	0.0119	0.0192
rEA	0.0313	0.0179	1.91 × 10^−3^	0.0109	0.0869	0.0142	0.0303
**tdYG1**	**101,162**	**103,842**	**7.61 × 10^8^**	**54,830**	**140,536**	**78,262**	**125,226**
**tdYG2**	**99,000**	**98,925**	**7.13 × 10^8^**	**55,038**	**137,970**	**76,482**	**124,250**
**tdOA1**	**77,106**	**73,566**	**5.86 × 10^8^**	**47,019**	**120,206**	**55,392**	**96,881**
tOAbot1	73,389	66,248	6.14 × 10^8^	44,341	118,942	52,082	93,165
**tdOA2**	**47,524**	**45,937**	**3.99 × 10^7^**	**40,394**	**59,245**	**42,597**	**51,019**
tOAbot2	45,223	43,282	5.30 × 10^7^	37,718	58,387	40,110	48,153
tdG2BE	68,415	61,497	3.78 × 10^8^	50,281	113,560	53,713	75,889
**tdEA**	**38,187**	**37,017**	**4.33 × 10^7^**	**30,483**	**50,076**	**33,374**	**41,444**
taNG2	52,032	49,731	8.13 × 10^7^	42,680	69,758	45,402	55,444
taNEA	41,663	40,005	4.51 × 10^7^	33,965	55,743	36,653	45,055
taARP	61,567	55,048	4.53 × 10^8^	37,831	106,642	43,945	75,654
taD1P	51,047	44,460	3.89 × 10^8^	31,094	95,155	36,207	58,088
taD2A	28,645	27,059	4.24 × 10^7^	20,958	39,746	23,730	32,456
taBEE	25,269	24,844	1.00 × 10^8^	11,194	45,254	16,827	31,380
paNG2	5.19 × 10^−2^	4.99 × 10^−2^	7.71 × 10^−4^	9.44 × 10^−3^	9.52 × 10^−3^	2.91 × 10^−2^	7.73 × 10^−2^
paNEA	4.73 × 10^−2^	4.73 × 10^−2^	7.95 × 10^−4^	5.36 × 10^−3^	9.57 × 10^−2^	2.30 × 10^−2^	7.01 × 10^−2^
paARP	4.82 × 10^−2^	4.83 × 10^−2^	9.00 × 10^−4^	4.97 × 10^−3^	9.45 × 10^−2^	2.09 × 10^−2^	7.71 × 10^−2^
paD1P	5.21 × 10^−2^	5.27 × 10^−2^	8.43 × 10^−4^	4.58 × 10^−3^	9.53 × 10^−2^	2.84 × 10^−2^	7.85 × 10^−2^
paD2A	4.74 × 10^−2^	4.72 × 10^−2^	8.46 × 10^−4^	3.95 × 10^−3^	9.32 × 10^−2^	2.17 × 10^−2^	7.24 × 10^−2^
paBEE	2.78 × 10^−1^	2.85 × 10^−1^	1.61 × 10^−2^	6.83 × 10^−2^	4.79 × 10^−1^	1.71 × 10^−1^	3.83 × 10^−1^
mYG1	4.75 × 10^−4^	4.62 × 10^−4^	9.64 × 10^−8^	2.61 × 10^−5^	9.48 × 10^−4^	1.92 × 10^−4^	7.54 × 10^−4^
mG1Y	4.74 × 10^−4^	4.64 × 10^−4^	7.95 × 10^−8^	4.65 × 10^−5^	9.30 × 10^−4^	2.25 × 10^−4^	6.98 × 10^−4^
mG1G2	4.93 × 10^−4^	4.80 × 10^−4^	8.50 × 10^−8^	4.54 × 10^−5^	9.41 × 10^−4^	2.49 × 10^−4^	7.63 × 10^−4^
mG2G1	5.34 × 10^−4^	5.61 × 10^−4^	8.83 × 10^−8^	4.77 × 10^−5^	9.68 × 10^−4^	2.69 × 10^−4^	7.94 × 10^−4^
mG2E	5.23 × 10^−4^	5.29 × 10^−4^	8.13 × 10^−8^	5.19 × 10^−5^	9.57 × 10^−4^	2.84 × 10^−4^	7.81 × 10^−4^
mEG2	4.21 × 10^−4^	3.69 × 10^−4^	7.78 × 10^−8^	3.73 × 10^−5^	9.07 × 10^−4^	1.85 × 10^−4^	6.48 × 10^−4^
mEA	4.19 × 10^−4^	3.60 × 10^−4^	8.63 × 10^−8^	3.73 × 10^−5^	9.66 × 10^−4^	1.81 × 10^−4^	6.45 × 10^−4^
mAE	5.33 × 10^−4^	5.69 × 10^−4^	7.63 × 10^−8^	5.82 × 10^−5^	9.33 × 10^−4^	2.90 × 10^−4^	7.57 × 10^−4^
mAP	1.70 × 10^−4^	1.27 × 10^−4^	2.26 × 10^−8^	1.42 × 10^−5^	5.16 × 10^−4^	7.40 × 10^−5^	2.10 × 10^−4^
mPA	1.28 × 10^−4^	1.02 × 10^−4^	1.18 × 10^−8^	8.01 × 10^−6^	3.37 × 10^−4^	4.52 × 10^−5^	1.72 × 10^−4^
m1G2EA	4.96 × 10^−4^	5.01 × 10^−4^	8.24 × 10^−8^	5.60 × 10^−6^	9.47 × 10^−4^	2.45 × 10^−4^	7.53 × 10^−4^
m1EAG2	4.46 × 10^−4^	4.00 × 10^−4^	8.23 × 10^−8^	5.18 × 10^−5^	9.49 × 10^−4^	1.99 × 10^−4^	6.95 × 10^−4^
m1EAP	4.25 × 10^−4^	3.97 × 10^−4^	7.57 × 10^−8^	2.77 × 10^−5^	9.07 × 10^−4^	1.95 × 10^−4^	6.39 × 10^−4^
m1PEA	4.40 × 10^−4^	4.02 × 10^−4^	8.39 × 10^−8^	4.04 × 10^−5^	9.31 × 10^−4^	1.77 × 10^−4^	6.93 × 10^−4^

**Table 4:** Estimated parameters for the MD model using the Papuan samples from [12]. The mean and the median estimated values are listed, as well as the 90% and the 50% credible intervals. The parameters cited in the text are reported in bold.

Parameter	Mean	Median	Variance	Q (0.05)	Q (0.95)	Q (0.25)	Q (0.75)
**nAR**	**2803**	**2783**	**4.57 × 10^4^**	**2532**	**3302**	**2668**	**2900**
**nY**	**19,182**	**14,771**	**1.62 × 10^8^**	**4379**	**44,930**	**8223**	**29,102**
nG1	26,722	28,003	2.18 × 10^8^	2702	47,514	14,075	40,579
nG2	25,325	27,394	1.97 × 10^8^	2218	47,188	13,362	36,308
nBE	25,684	26,296	2.17 × 10^8^	2194	47,896	13,706	38,919
**nE**	**12,485**	**5373**	**1.94 × 10^8^**	**699**	**42,194**	**1616**	**21,836**
**nA**	**14,543**	**8978**	**2.10 × 10^8^**	**916**	**43,930**	**2214**	**26,207**
**nP**	**19,089**	**16,639**	**2.16 × 10^8^**	**1048**	**46,319**	**4980**	**30,429**
**nYG**	**22,857**	**21,922**	**2.62 × 10^7^**	**17,112**	**31,789**	**19,579**	**25,130**
**nNNR**	**2422**	**2336**	**1.24 × 10^5^**	**2057**	**3023**	**2219**	**2531**
nDDR	21,778	20,572	1.94 × 10^8^	1640	46291	9606	32,332
nDN	16,239	11,846	1.59 × 10^8^	2879	41321	5311	25,523
nADN	19,279	16,531	2.21 × 10^8^	2108	47070	4884	31,082
**nAM**	**18,629**	**18,574**	**1.57 × 10^6^**	**16,671**	**20,691**	**17,779**	**19,476**
rP	0.0215	0.0143	6.10 × 10^−4^	0.0104	0.0576	0.0118	0.0204
rEA	0.0314	0.0179	1.94 × 10^−3^	0.0109	0.0869	0.0144	0.0310
**tdYG1**	**98,829**	**99,987**	**7.31 × 10^8^**	**54,220**	**140,009**	**76,337**	**122,428**
**tdYG2**	**97,430**	**96,686**	**6.87 × 10^8^**	**54,693**	**138,490**	**76,482**	**120,370**
**tdOA1**	**74,244**	**68,987**	**5.32 × 10^8^**	**46,663**	**119,539**	**54,334**	**89,685**
tOAbot1	70,341	64,285	5.47 × 10^8^	43,471	116,608	50,992	85,938
**tdOA2**	**48,554**	**46,257**	**7.36 × 10^7^**	**40,559**	**64,865**	**42,739**	**51,453**
tOAbot2	46,366	43,475	8.49 × 10^7^	37,922	63,074	40,247	50,084
tdG2BE	68,122	62,035	3.36 × 10^8^	50,281	105,774	53,533	76,526
**tdEA**	**37,747**	**35,936**	**5.05 × 10^7^**	**30,381**	**50,399**	**32,690**	**40,845**
taNG2	53,606	50,116	1.08 × 10^8^	43,274	73,012	46,917	57,484
taNEA	42,255	40,175	7.98 × 10^7^	33,449	56,376	37,030	45,231
taARP	61,203	54,697	4.60 × 10^8^	37,428	106,643	43,994	73,444
taD1P	48,493	43,651	2.90 × 10^8^	31,343	86,579	36,450	55,023
taD2A	29,298	27,601	5.05 × 10^7^	21,090	41,451	24,133	32,700
taBEE	23,871	23,356	9.64 × 10^7^	10,508	40,711	15,268	30,666
paNG2	5.29 × 10^−2^	5.35 × 10^−2^	7.32 × 10^−4^	8.94 × 10^−3^	9.52 × 10^−2^	3.18 × 10^−2^	7.51 × 10^−2^
paNEA	5.12 × 10^−2^	5.22 × 10^−2^	7.83 × 10^−4^	5.58 × 10^−3^	9.60 × 10^−2^	2.69 × 10^−2^	7.44 × 10^−2^
paARP	5.02 × 10^−2^	5.06 × 10^−2^	8.74 × 10^−4^	5.45 × 10^−3^	9.49 × 10^−2^	2.36 × 10^−2^	7.81 × 10^−2^
paD1P	5.23 × 10^−2^	5.50 × 10^−2^	8.00 × 10^−4^	6.13 × 10^−3^	9.41 × 10^−2^	2.78 × 10^−2^	7.66 × 10^−2^
paD2A	4.82 × 10^−2^	4.52 × 10^−2^	8.87 × 10^−4^	4.93 × 10^−3^	9.58 × 10^−2^	2.27 × 10^−2^	7.39 × 10^−2^
paBEE	2.79 × 10^−1^	2.91 × 10^−1^	1.65 × 10^−2^	6.58 × 10^−2^	4.78 × 10^−1^	1.68 × 10^−1^	3.88 × 10^−1^
mYG1	4.47 × 10^−4^	4.08 × 10^−4^	8.52 × 10^−8^	3.74 × 10^−5^	9.32 × 10^−4^	1.89 × 10^−4^	6.97 × 10^−4^
mG1Y	4.92 × 10^−4^	4.91 × 10^−4^	7.55 × 10^−8^	5.11 × 10^−5^	9.27 × 10^−4^	2.79 × 10^−4^	7.28 × 10^−4^
mG1G2	4.74 × 10^−4^	4.59 × 10^−4^	8.40 × 10^−8^	4.41 × 10^−5^	9.35 × 10^−4^	2.31 × 10^−4^	7.32 × 10^−4^
mG2G1	5.20 × 10^−4^	5.23 × 10^−4^	9.07 × 10^−8^	4.77 × 10^−5^	9.67 × 10^−4^	2.34 × 10^−4^	7.93 × 10^−4^
mG2E	5.16 × 10^−4^	5.29 × 10^−4^	7.87 × 10^−8^	5.67 × 10^−5^	9.55 × 10^−4^	2.85 × 10^−4^	7.60 × 10^−4^
mEG2	3.77 × 10^−4^	3.04 × 10^−4^	8.13 × 10^−8^	2.70 × 10^−5^	9.11 × 10^−4^	1.30 × 10^−4^	5.80 × 10^−4^
mEA	5.07 × 10^−4^	5.15 × 10^−4^	8.78 × 10^−8^	4.74 × 10^−5^	9.57 × 10^−4^	2.52 × 10^−4^	7.68 × 10^−4^
mAE	4.67 × 10^−4^	4.68 × 10^−4^	7.94 × 10^−8^	4.78 × 10^−5^	9.17 × 10^−4^	2.29 × 10^−4^	7.07 × 10^−4^
mAP	5.17 × 10^−4^	5.12 × 10^−4^	7.28 × 10^−8^	1.04 × 10^−4^	9.35 × 10^−4^	2.78 × 10^−4^	7.50 × 10^−4^
mPA	4.05 × 10^−4^	3.79 × 10^−4^	5.71 × 10^−8^	5.15 × 10^−5^	8.70 × 10^−4^	2.27 × 10^−4^	5.41 × 10^−4^
m1G2EA	5.20 × 10^−4^	5.21 × 10^−4^	8.85 × 10^−8^	4.88 × 10^−5^	9.74 × 10^−4^	2.74 × 10^−4^	7.90 × 10^−4^
m1EAG2	4.56 × 10^−4^	4.30 × 10^−4^	7.91 × 10^−8^	5.77 × 10^−5^	9.24 × 10^−4^	2.09 × 10^−4^	7.16 × 10^−4^
m1EAP	4.92 × 10^−4^	5.12 × 10^−4^	7.88 × 10^−8^	6.32 × 10^−5^	9.42 × 10^−4^	2.47 × 10^−4^	7.11 × 10^−4^
m1PEA	4.78 × 10^−4^	4.59 × 10^−4^	7.42 × 10^−8^	6.17 × 10^−5^	9.24 × 10^−4^	2.44 × 10^−4^	7.02 × 10^−4^

**Table 5:** Accuracy of the estimated parameters of the MD model assessed by 1000 pods. The parameters cited in the text are reported in bold.

Parameters	R^2^	Bias	RMSE	Factor 2	Coverage 90%	Coverage 50%
**nAR**	**0.84**	**−0.0020**	**5.90 × 10^3^**	**0.990**	**0.935**	**0.553**
**nY**	**0.54**	**0.1900**	**1.04 × 10^4^**	**0.867**	**0.919**	**0.522**
nG1	0.08	2.0020	1.46 × 10^4^	0.702	0.880	0.466
nG2	0.17	0.9175	1.36 × 10^4^	0.698	0.915	0.497
nBE	0.02	2.2194	1.47 × 10^4^	0.722	0.895	0.479
**nE**	**0.33**	**0.4278**	**1.25 × 10^4^**	**0.767**	**0.908**	**0.523**
**nA**	**0.28**	**0.4159**	**1.20 × 10^4^**	**0.795**	**0.922**	**0.532**
**nP**	**0.39**	**0.3425**	**1.21 × 10^4^**	**0.791**	**0.908**	**0.501**
**nYG**	**0.91**	**0.0020**	**3.54 × 10^3^**	**0.998**	**0.957**	**0.650**
**nNNR**	**0.92**	**0.0086**	**3.64 × 10^3^**	**0.998**	**0.966**	**0.622**
nDDR	0.36	0.3529	1.18 × 10^4^	0.800	0.923	0.522
nDN	0.54	0.1979	1.09 × 10^4^	0.842	0.941	0.534
nADN	0.33	0.7749	1.29 × 10^4^	0.705	0.930	0.476
**nAM**	**0.99**	**0.0067**	**5.40 × 10^2^**	**0.997**	**0.995**	**0.870**
rP	0.10	0.1110	6.79 × 10^−2^	0.721	0.879	0.521
rEA	0.10	0.0983	5.65 × 10^−2^	0.748	0.915	0.547
**tdYG1**	**0.25**	**0.0629**	**2.23 × 10^4^**	**0.998**	**0.928**	**0.576**
**tdYG2**	**0.25**	**0.0630**	**2.25 × 10^4^**	**0.996**	**0.934**	**0.573**
**tdOA1**	**0.19**	**0.0025**	**1.99 × 10^4^**	**0.998**	**0.911**	**0.540**
tOAbot1	0.19	0.0052	1.99 × 10^4^	0.996	0.918	0.544
**tdOA2**	**0.13**	**−0.0257**	**1.24 × 10^4^**	**0.998**	**0.883**	**0.511**
tOAbot2	0.13	−0.0261	1.24 × 10^4^	0.995	0.881	0.512
tdG2BE	0.16	−0.0016	1.98 × 10^4^	0.999	0.913	0.523
**tdEA**	**0.08**	**−0.0167**	**9.09 × 10^3^**	**0.989**	**0.898**	**0.495**
taD2A	0.04	0.0116	7.35 × 10^3^	0.993	0.905	0.526
paD2A	0.02	0.0010	2.88 × 10^−2^	1.000	0.900	0.500
taBEE	0.03	0.1286	1.04 × 10^4^	0.914	0.904	0.486
paBEE	0.02	0.0439	1.31 × 10^−1^	1.000	0.893	0.497
taD1P	0.11	−0.0070	1.72 × 10^4^	0.973	0.897	0.499
paD1P	0.02	−0.0002	2.85 × 10^−2^	1.000	0.897	0.508
taARP	0.15	−0.0002	1.85 × 10^4^	0.988	0.916	0.517
paARP	0.03	−0.0014	2.85 × 10^−2^	1.000	0.906	0.509
taNEA	0.10	−0.0204	1.06 × 10^4^	0.992	0.893	0.516
paNEA	0.02	0.0000	2.81 × 10^−2^	1.000	0.924	0.516
taNG2	0.15	−0.0223	1.36 × 10^4^	0.998	0.909	0.528
paNG2	0.02	−0.0003	2.89 × 10^−2^	1.000	0.909	0.477
mYG1	0.15	1.2696	2.69 × 10^−4^	0.709	0.927	0.521
mG1Y	0.03	1.8171	2.86 × 10^−4^	0.742	0.907	0.516
mG1G2	0.05	2.0667	2.85 × 10^−4^	0.737	0.895	0.519
mG2G1	0.05	2.9954	2.89 × 10^−4^	0.745	0.885	0.509
mG2E	0.03	3.0547	3.01 × 10^−4^	0.692	0.886	0.460
mEG2	0.19	1.5013	2.67 × 10^−4^	0.722	0.908	0.503
mEA	0.12	1.4834	2.68 × 10^−4^	0.744	0.902	0.543
mAE	0.11	1.9813	2.74 × 10^−4^	0.731	0.908	0.523
mAP	0.27	1.4789	2.40 × 10^−4^	0.766	0.910	0.548
mPA	0.37	2.2687	2.35 × 10^−4^	0.773	0.908	0.546
m1G2EA	0.02	2.1201	2.90 × 10^−4^	0.701	0.911	0.489
m1EAG2	0.04	2.7879	2.92 × 10^−4^	0.708	0.888	0.496
m1EAP	0.06	2.5111	2.82 × 10^−4^	0.728	0.901	0.528
m1PEA	0.05	3.2113	2.91 × 10^−4^	0.694	0.911	0.477

## Supplementary Materials

The following are available online at https://www.mdpi.com/2073-4425/11/12/1510/s1, Table S1: Demographic parameters and prior distributions of Single Dispersal model. Table S2: Demographic parameters and prior distributions of Multiple Dispersal model. Table S3: Complete list of genomes used for the comparison of Single Dispersal model and Multiple Dispersal model using real data; Table S4: Results of model selection performed using alternative individuals from African, European and Asian populations; Table S5: Power test of model comparison for increasing number of simulations considered in the reference table.; Table S6. Complete list of acronyms of the MD model’s demographic parameters.; Table S7. Model Selection results including the MD-Pulse admixture model. Figure S1: Outline of the entire workflow; Figure S2: Posterior density of the effective population sizes estimated using the Papuan sample from Malaspinas et al. (2016). Figure S3: Posterior density of the divergence times and the admixture times estimated using the Papuan sample from Malaspinas et al. (2016). Figure S4: Posterior density of the admixture rates estimated using the Papuan sample from Malaspinas et al. (2016). Figure S5: Posterior density of the migration rates estimated using the Papuan sample from Malaspinas et al. (2016). Figure S6: Posterior density of the effective population sizes estimated using the Papuan sample from Pagani et al. (2016). Figure S7: Posterior density of the divergence times and the admixture times estimated using the Papuan sample from Pagani et al. (2016). Figure S8: Posterior density of the admixture rates estimated using the Papuan sample from Pagani et al. (2016). Figure S9: Posterior density of the migration rates estimated using the Papuan sample from Pagani et al. (2016). Figure S10: The model below represents a simplified version of the most supported model (MD) showing the main demographic parameters.
