# Supplementary material for: A Revised Model of Anatomically Modern Human Expansions Out of Africa through a Machine Learning Approximate Bayesian Computation Approach
Source: Genes (Basel). 2020 Dec 16;11(12):1510. doi: 10.3390/genes11121510 (PMC7766041; doi:10.3390/genes11121510)

Table S1. Demographic parameters and prior distributions of Single Dispersal model. Migration and admixture rates are expressed per generation, times in years. We considered a generation time of 29 years as in Malaspinas et al. (2016). Per nucleotide per generation mutation and recombination rates are fixed as in Malaspinas et al. (2016). Parameters defined by prior distributions having the same shape and range are indicated through the same entry.

Demographic Parameters	Prior Distributions
Effective population size (Ne)	Uniform {500:50,000}
Migration rate (ModernPop)	Uniform {10^{-6}: 10^{-3}}
Time split Africa-Ghost	Uniform {50,000:145,000}yrs
Duration time bottleneck	2,900yrs
Intensity bottleneck	Uniform {2:100}
Time split African Ghost – BasalEurope	EndBottleneck African Ghost yrs
Time split Eurasia/Papua-Ghost(OOA)	Uniform {45,000:EndBottlGhost}yrs
Time split Europe-Asia	Uniform {30,000: EndbottlOOA }yrs
Time admixture Nea-Eurasia	Uniform {Time split Europe-Asia:EndbottlOOA}yrs
Time admixture Den-Papua	Uniform {30,000:EndBottlOOA}yrs
Time admixture Den2-Asia	Uniform {20,000:Time split Europe-Asia}yrs
Time admixture Arc-Papua	Uniform {Time admix. Den-Papua: EndBottl.OOA}yrs
Time admixture Nea-Ghost	Uniform {Time split. Eurs/Pap-Ghost:EndBottl.Ghost}yrs
Time admixture Basal Europe - Europe	Uniform {10,000:Time split Europe-Asia}yrs
Admixture rate (Archaic–Modern pop)	Uniform {10^{-3}:10^{-1}}
Admixture rate (BasalEurope–Europe)	Uniform {5%-50%}
Time split Nea-NeaR	110,000yrs {Fixed}
Time split Den-DenR	393,000yrs {Fixed}
Time split Den-Nea	495,000yrs {Fixed}
Time split Arc-Nea/Den	580,000yrs {Fixed}
Time split Ancient-Modern	638,000yrs {Fixed}
Sample Time Neandertal	85,735yrs {Fixed}
Sample Time Denisova	67,570yrs {Fixed}
Mutation rate	1.25×10^{-8} {Fixed}
Recombination rate	1.12×10^{-8} {Fixed}

Table S2. Demographic parameters and prior distributions of Multiple Dispersal model. Migration and admixture rates are expressed per generation, times in years. We cosidered a generation time of 29 years as in Malaspinas et al. (2016). Per nucleotide per generation mutation and recombination rates are fixed as in Malaspinas et al. (2016). Parameters defined by prior distributions having the same shape and range are indicated through the same entry.

Demographic Parameters	Prior Distributions
Effective population size (Ne)	Uniform {500:50.000}
Migration rate (ModerPop)	Uniform {10^{-6}: 10^{-3}}
Time split Africa-Ghosts(1 and 2)	Uniform {50,000:145,000}yrs
Duration time bottleneck	2,900yrs
Intensity bottleneck	Uniform {2:100}
Time split Ghost2-BasalEurope	Uniform {50,000:Time split. Africa-Ghosts}yrs
Time split Papua-Ghost1(OOA1)	Uniform {45,000:Time split. Africa-Ghost1}yrs
Time split Eurasia-Ghost2(OOA2)	Uniform {40,000:EndBott.OOA1}yrs
Time split Europe-Asia	Uniform {30,000:EndBott.OOA2}yrs
Time admixture Nea-Eurasia	Uniform {Time split Europe-Asia:EndBott.OOA2}yrs
Time admixture Den-Papua	Uniform {30,000: EndBott.OOA1}yrs
Time admixture Den2-Asia	Uniform {20,000:Time split Europe-Asia}yrs

Time admixture BasalEurope-Europe	Uniform {10,000:Time split Europe-Asia}yrs
Time admixture Arc-Papua	Uniform {Time admix. Den-Papua:EndBott.OOA1}yrs
Time admixture Nea-Ghost2	Uniform {Time split Euras-Ghost2:Time split Africa-Ghost2}yrs
Admixture rate (Archaic–Modern pop)	Uniform {10^{-3}:10^{-1}}
Admixture rate (BasalEurope–Europe)	Uniform {5%-50%}
Time split Nea-NeaR	110,000yrs {Fixed}
Time split Den-DenR	393,000yrs {Fixed}
Time split Den-Nea	495,000yrs {Fixed}
Time split Arc-Nea/Den	580,000yrs {Fixed}
Time split Ancient-Modern	638,000yrs {Fixed}
Sample Time Neandertal	85,735yrs {Fixed}
Sample Time Denisova	67,570yrs {Fixed}
Mutation rate	$1.25x10^{-8}$ {Fixed}
Recombination rate	$1.12x10^{-8}$ {Fixed}

Table S3. Complete list of genomes used for the comparison of Single Dispersal model and Multiple Dispersal model using real data.

Population	ID_Individual	Reference
Neandertal	AltaiNea	Prufer *et al.* (2014)
Denisova	DenisovaPinky	Mayer *et al.* (2012)
African	CongPy1	Pagani *et al.* (2016)
African	CongPy3	Pagani *et al.* (2016)
African	CongPy6	Pagani *et al.* (2016)
European	Est1	Pagani *et al.* (2016)
European	Est2	Pagani *et al.* (2016)
European	Est3	Pagani *et al.* (2016)
European	Est4	Pagani *et al.* (2016)
European	Est5	Pagani *et al.* (2016)
European	Est6	Pagani *et al.* (2016)
Asian	VietN1	Pagani *et al.* (2016)
Asian	VietN2	Pagani *et al.* (2016)
Asian	VietC1	Pagani *et al.* (2016)
Asian	VietC2	Pagani *et al.* (2016)
Asian	VietS1	Pagani *et al.* (2016)
Asian	VietS2	Pagani *et al.* (2016)
Papuan	Koinb1	Pagani *et al.* (2016)
Papuan	Koinb2	Pagani *et al.* (2016)
Papuan	Koinb3	Pagani *et al.* (2016)
Papuan	Kosip1	Pagani *et al.* (2016)
Papuan	Kosip2	Pagani *et al.* (2016)
Papuan	Kosip3	Pagani *et al.* (2016)
Papuan	EGAN00001279031	Malaspinas *et al.* (2016)
Papuan	EGAN00001279039	Malaspinas *et al.* (2016)
Papuan	EGAN00001279047	Malaspinas *et al.* (2016)
Papuan	EGAN00001279054	Malaspinas *et al.* (2016)
Papuan	EGAN00001279032	Malaspinas *et al.* (2016)
Papuan	EGAN00001279040	Malaspinas *et al.* (2016)
Papuan	EGAN00001279048	Malaspinas *et al.* (2016)
Papuan	EGAN00001279033	Malaspinas *et al.* (2016)
Papuan	EGAN00001279041	Malaspinas *et al.* (2016)

Papuan	EGAN00001279049	Malaspinas *et al.* (2016)
Papuan	EGAN00001279034	Malaspinas *et al.* (2016)
Papuan	EGAN00001279042	Malaspinas *et al.* (2016)
Papuan	EGAN00001279050	Malaspinas *et al.* (2016)
Papuan	EGAN00001279035	Malaspinas *et al.* (2016)
Papuan	EGAN00001279043	Malaspinas *et al.* (2016)
Papuan	EGAN00001279051	Malaspinas *et al.* (2016)
Papuan	EGAN00001279036	Malaspinas *et al.* (2016)
Papuan	EGAN00001279044	Malaspinas *et al.* (2016)
Papuan	EGAN00001279052	Malaspinas *et al.* (2016)
Papuan	EGAN00001279037	Malaspinas *et al.* (2016)
Papuan	EGAN00001279045	Malaspinas *et al.* (2016)
Papuan	EGAN00001279053	Malaspinas *et al.* (2016)
Papuan	EGAN00001279038	Malaspinas *et al.* (2016)
Papuan	EGAN00001279046	Malaspinas *et al.* (2016)
Papuan	EGAN00001279055	Malaspinas *et al.* (2016)

Table S4. Results of model selection performed using alternative individual from African, European and Asian populations.

ID_Individual	Selected model	Votes SD	Votes MD	Post.Prob.
CongPy3	MD	152	348	0.83
CongPy6	MD	167	333	0.81
Est2	MD	120	380	0.82
Est3	MD	113	387	0.80
Est4	MD	132	368	0.81
Est5	MD	108	392	0.82
Est6	MD	181	319	0.80
VietN2	MD	111	389	0.83
VietC1	MD	100	400	0.84
VietC2	MD	153	347	0.84
VietS1	MD	145	355	0.83
VietS2	MD	150	350	0.82

Table S5. Power test of model comparison for increasing number of simulations considered in the reference table.

Prior Err. Rate	True Positive SD	True Positive MD	Post.Prob. SD	Post.Prob. MD	n. Sim.
0.271	0.720	0.736	0.733	0.724	20,000
0.264	0.723	0.748	0.745	0.724	50,000
0.260	0.730	0.755	0.750	0.732	100,000

Table S6. Complete list of acronyms of the MD model's demographic parameters.

Acronym	Parameters
nAR	Effective population size UnknownArchaic
nY	Effective population size Africa
nG1	Effective population size Ghost1
nG2	Effective population size Ghost2
nBE	Effective population size Basal Europe
nE	Effective population size Europe
nA	Effective population size Asia

nP	Effective population size Papua
nYG	Effective population size Ancestral Africa
nNNR	Effective population size Ancestral Neandertal
nDDR	Effective population size Ancestral Denisovan
nDN	Effective population size Ancestral Denisova-Neandertal population
nADN	Effective population size Ancestral Archaic populations
nAM	Effective population size Ancestral Archaic-Modern population
rP	Intensity Bottleneck Papua
rEA	Intensity Bottleneck Eurasia
tdYG1	Divergence time African-Ghost populations
tdYG2	Divergence time African-Ghost populations
tdOA1	Time of the first Out-of-Africa
tOAbot1	Time end bottleneck first Out-of Africa
tdOA2	Time of the second Out-of-Africa
tOAbot2	Time end bottleneck second Out-of Africa
tdG2BE	Divergence time Africa-Basal Europe
tdEA	Divergence time Europe-Asia
taD2A	Admixture time Denisova2-Asia
paD2A	Admixture rate Denisova2-Asia
taBEE	Admixture time Basal Europe-Europe
paBEE	Admixture rate Basal Europe-Europe
taD1P	Admixture time Denisova1-Papua
paD1P	Admixture rate Denisova1-Papua
taARP	Admixture time UnknownArchaic-Papua
paARP	Admixture rate UnknownArchaic-Papua
taNEA	Admixture time Neandertal- Eurasia
paNEA	Admixture rate Neandertal- Eurasia
taNG2	Admixture time Neandertal- Ghost2
paNG2	Admixture rate Neandertal- Ghost2
mYG1	Migration rate Africa-Ghost1
mG1Y	Migration rate Ghost1-Africa
mG1G2	Migration rate Ghost1- Ghost2
mG2G1	Migration rate Ghost2- Ghost1
mG2E	Migration rate Ghost2-Europe
mEG2	Migration rate Europe-Ghost2
mEA	Migration rate Europe-Asia
mAE	Migration rate Asia-Europe
mAP	Migration rate Asia-Papua
mPA	Migration rate Papua-Asia
m1G2EA	Migration rate Ghost2-Eurasia
m1EAG2	Migration rate Eurasia-Ghost2
m1EAP	Migration rate Eurasia-Papua
m1PEA	Migration rate Papua-Eurasia

Table S7. Model Selection results including the MD-Pulse admixture model. In the first column are reported the ID of the Papuan samples used for the model choice. The second column shows the model selected by the ABC procedure. In the third, fourth and fifth columns are reported the votes assigned to the SD, the MD-Continuous migration and the MD- Pulse Admixture models by the Random-Forest algorithm. The last column shows the posterior probabilities associated to the most supported model.

ID_Individual	Selected model	Votes SD	Votes MD-Cont.Migration	Votes MD-PulseAdmx	Post.Prob.
EGAN00001279031	MD- Cont.Migration	68	235	197	0.81
EGAN00001279039	MD- PulseAdmx	50	191	259	0.82
EGAN00001279047	MD- PulseAdmx	45	212	243	0.83
EGAN00001279054	MD- PulseAdmx	34	161	305	0.82
EGAN00001279032	MD- PulseAdmx	53	195	252	0.80
EGAN00001279040	MD- PulseAdmx	39	190	271	0.83
EGAN00001279048	MD- Cont.Migration	70	247	183	0.82
EGAN00001279033	MD- Cont.Migration	73	234	193	0.83
EGAN00001279041	MD- Cont.Migration	71	247	182	0.83
EGAN00001279049	MD- Cont.Migration	65	218	217	0.83
EGAN00001279034	MD- PulseAdmx	40	177	283	0.82
EGAN00001279042	MD- PulseAdmx	43	193	264	0.84
EGAN00001279050	MD- PulseAdmx	55	203	242	0.82
EGAN00001279035	MD- PulseAdmx	29	165	306	0.82
EGAN00001279043	MD- Cont.Migration	65	238	197	0.82
EGAN00001279051	MD- PulseAdmx	36	164	300	0.81
EGAN00001279036	MD- PulseAdmx	41	171	288	0.82
EGAN00001279044	MD- Cont.Migration	66	250	184	0.83
EGAN00001279052	MD- Cont.Migration	55	249	196	0.83
EGAN00001279037	MD- Cont.Migration	72	231	197	0.81
EGAN00001279045	MD- Cont.Migration	65	233	202	0.82
EGAN00001279053	MD- PulseAdmx	54	214	232	0.81
EGAN00001279038	MD- PulseAdmx	37	205	258	0.84
EGAN00001279046	MD- Cont.Migration	70	242	188	0.82
EGAN00001279055	MD- PulseAdmx	25	149	326	0.82
Koinb1	MD- Cont.Migration	120	298	82	0.80
Koinb2	MD- Cont.Migration	123	294	83	0.80
Koinb3	MD- Cont.Migration	135	269	96	0.82
Kosip1	MD- Cont.Migration	117	289	94	0.80
Kosip2	MD- Cont.Migration	106	294	100	0.81
Kosip3	MD- Cont.Migration	112	312	76	0.80

Figure S1: Outline of the entire workflow.

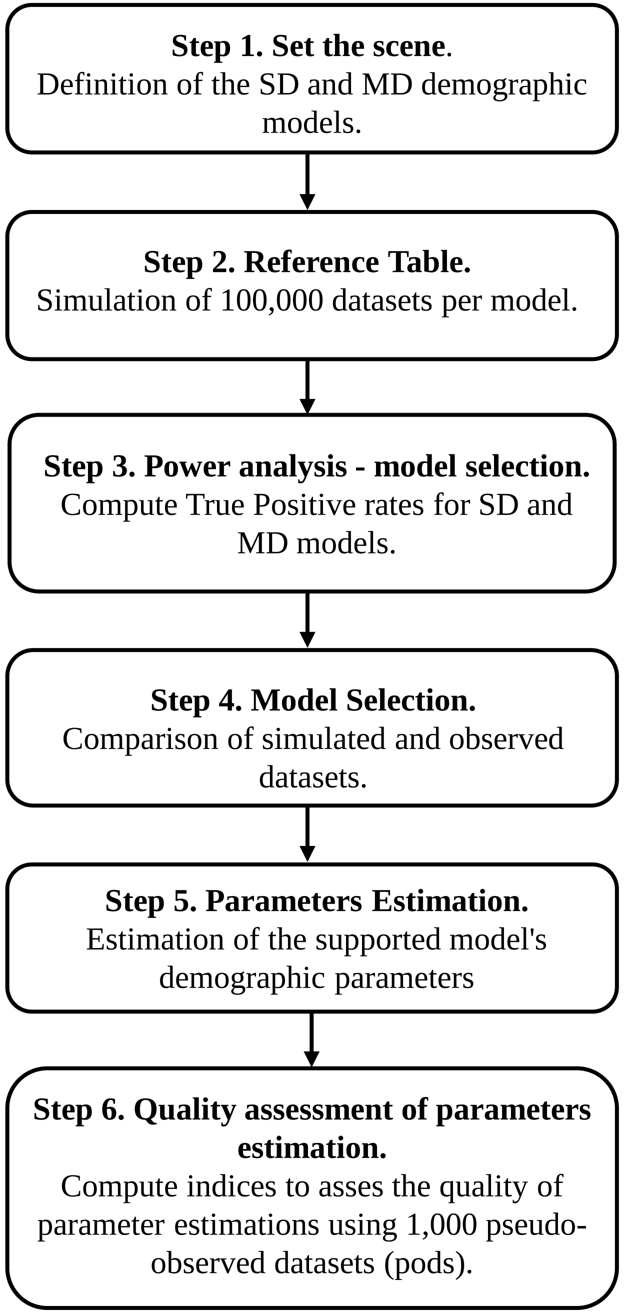
Step 1. Set the scene.
Definition of the SD and MD demographic models.
Step 2. Reference Table.
Simulation of 100,000 datasets per model.
Step 3. Power analysis - model selection.
Compute True Positive rates for SD and MD models.
Step 4. Model Selection.
Comparison of simulated and observed datasets.
Step 5. Parameters Estimation.
Estimation of the supported model's demographic parameters
Step 6. Quality assessment of parameters estimation.
Compute indices to asses the quality of parameter estimations using 1,000 pseudo-observed datasets (pods).

Figure S2: **Posterior density of the effective population sizes estimated using the Papuan sample from Malaspinas et al. (2016)**. The plots show: the posterior density (black), the mean (red) and median (blue) estimated values and the distribution of parameter's values sampled from the prior (gray).

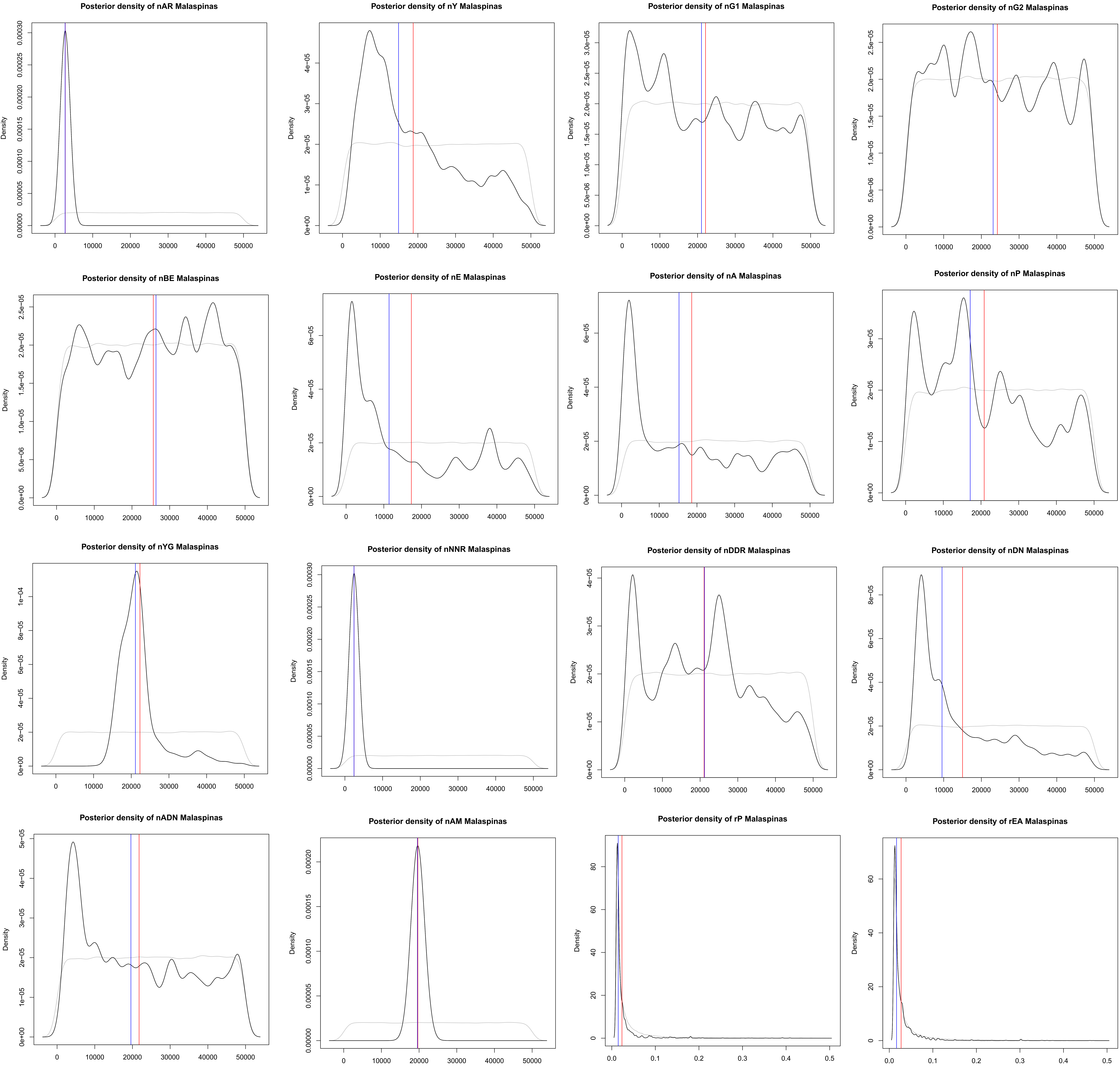

Posterior density of nAR Malaspinas
Posterior density of nY Malaspinas
Posterior density of nG1 Malaspinas
Posterior density of nG2 Malaspinas
Posterior density of nBE Malaspinas
Posterior density of nE Malaspinas
Posterior density of nA Malaspinas
Posterior density of nP Malaspinas
Posterior density of nYG Malaspinas
Posterior density of nNNR Malaspinas
Posterior density of nDDR Malaspinas
Posterior density of nDN Malaspinas
Posterior density of nADN Malaspinas
Posterior density of nAM Malaspinas
Posterior density of rP Malaspinas
Posterior density of rEA Malaspinas
Density
0
10000
20000
30000
40000
50000
0.0
0.1
0.2
0.3
0.4
0.5

Figure S3. **Posterior density of the divergence times and the admixture times estimated using the Papuan sample from Malaspinas et al. (2016)**. The plots have the same features of Figure S2.

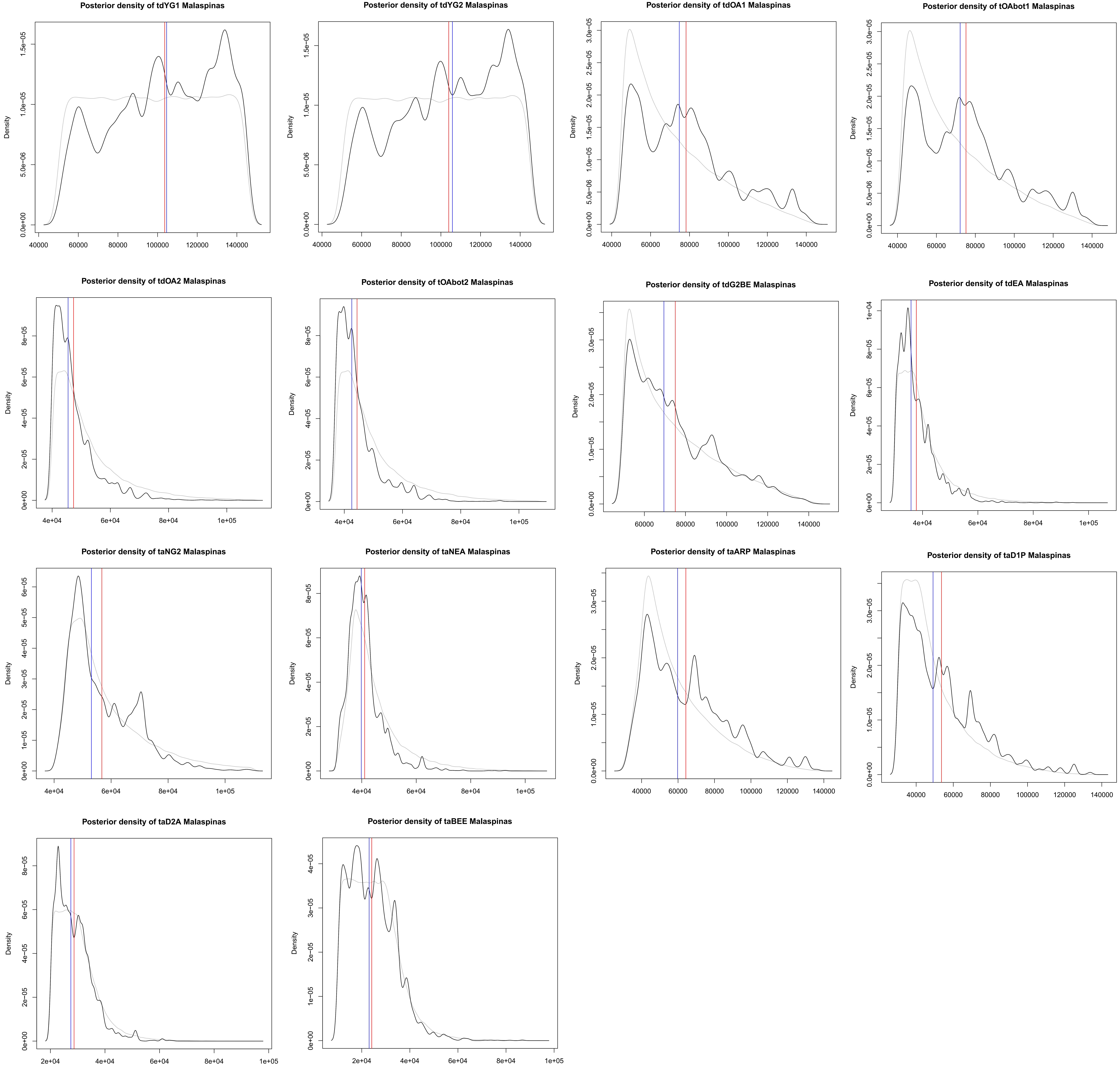

Posterior density of tdYG1 Malaspinas
Posterior density of tdYG2 Malaspinas
Posterior density of tdOA1 Malaspinas
Posterior density of tOAbot1 Malaspinas
Posterior density of tdOA2 Malaspinas
Posterior density of tOAbot2 Malaspinas
Posterior density of tdG2BE Malaspinas
Posterior density of tdEA Malaspinas
Posterior density of taNG2 Malaspinas
Posterior density of taNEA Malaspinas
Posterior density of taARP Malaspinas
Posterior density of taD1P Malaspinas
Posterior density of taD2A Malaspinas
Posterior density of taBEE Malaspinas
Density

Figure S4. **Posterior density of the admixture rates estimated using the Papuan sample from Malaspinas et al. (2016)**. The plots have the same features of Figure S2.

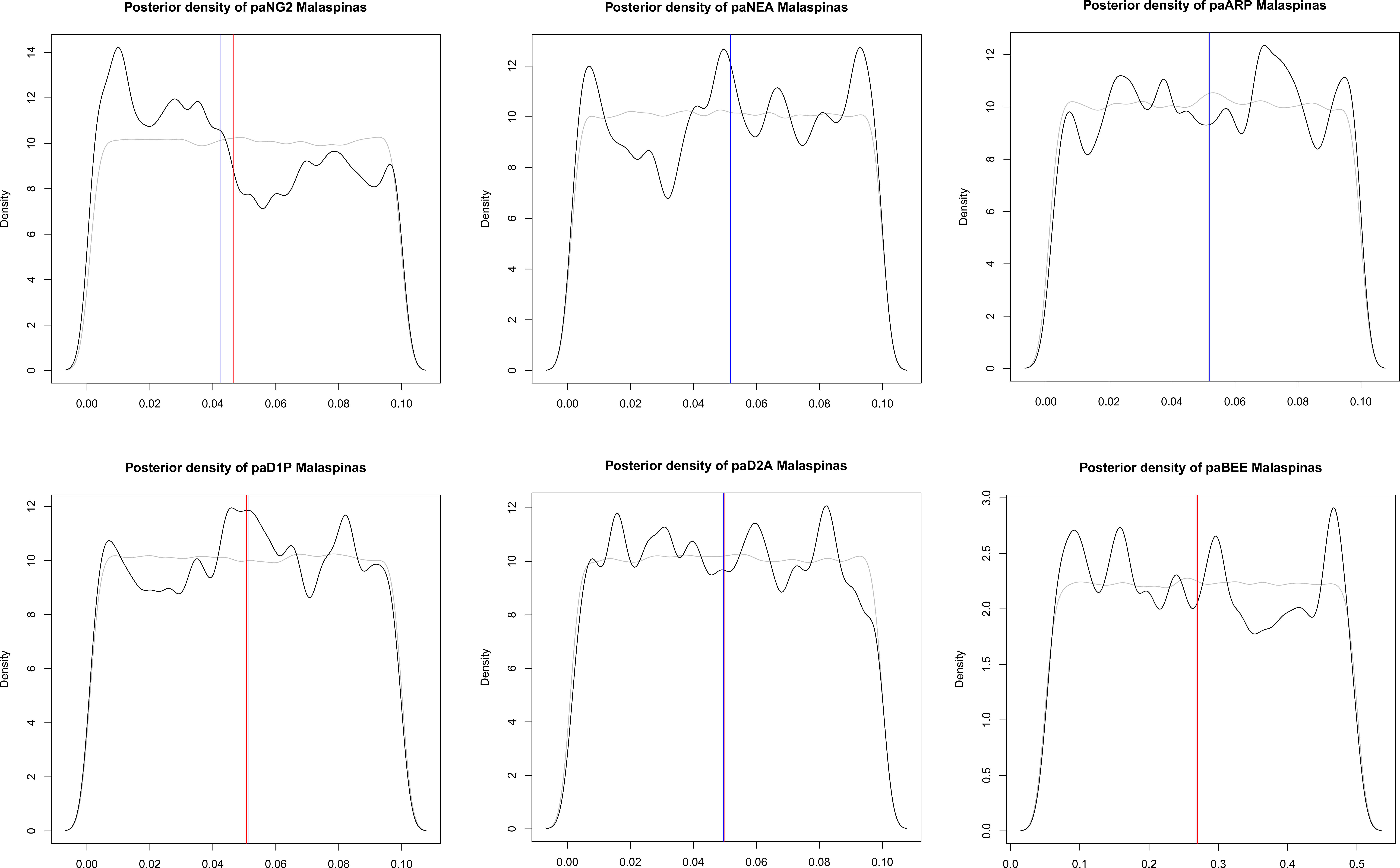
Posterior density of paNG2 Malaspinas
Posterior density of paNEA Malaspinas
Posterior density of paARP Malaspinas
Posterior density of paD1P Malaspinas
Posterior density of paD2A Malaspinas
Posterior density of paBEE Malaspinas
Density
0.00 0.02 0.04 0.06 0.08 0.10
0.0 0.1 0.2 0.3 0.4 0.5

Figure S5. Posterior density of the migration rates estimated using the Papuan sample from Malaspinas et al. (2016). The plots have the same features of Figure S2.

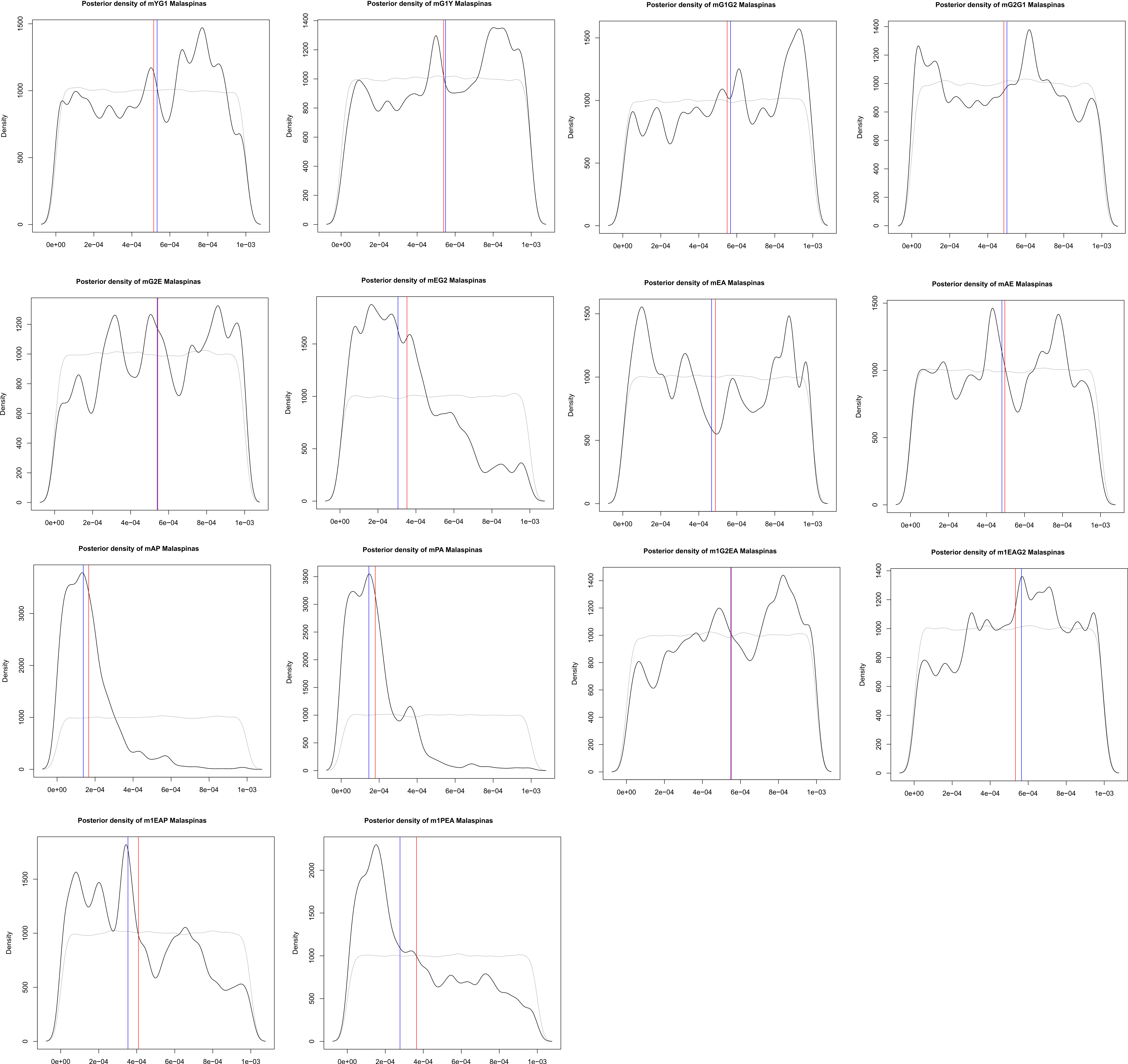
Posterior density of mYG1 Malaspinas
Posterior density of mG1Y Malaspinas
Posterior density of mG1G2 Malaspinas
Posterior density of mG2G1 Malaspinas
Posterior density of mG2E Malaspinas
Posterior density of mEG2 Malaspinas
Posterior density of mEA Malaspinas
Posterior density of mAE Malaspinas
Posterior density of mAP Malaspinas
Posterior density of mPA Malaspinas
Posterior density of m1G2EA Malaspinas
Posterior density of m1EAG2 Malaspinas
Posterior density of m1EAP Malaspinas
Posterior density of m1PEA Malaspinas
Density
0e+00
2e-04
4e-04
6e-04
8e-04
1e-03

Figure S6. **Posterior density of the effective population sizes estimated using the Papuan sample from Pagani et al. (2016)**. The plots have the same features of Figure S2.

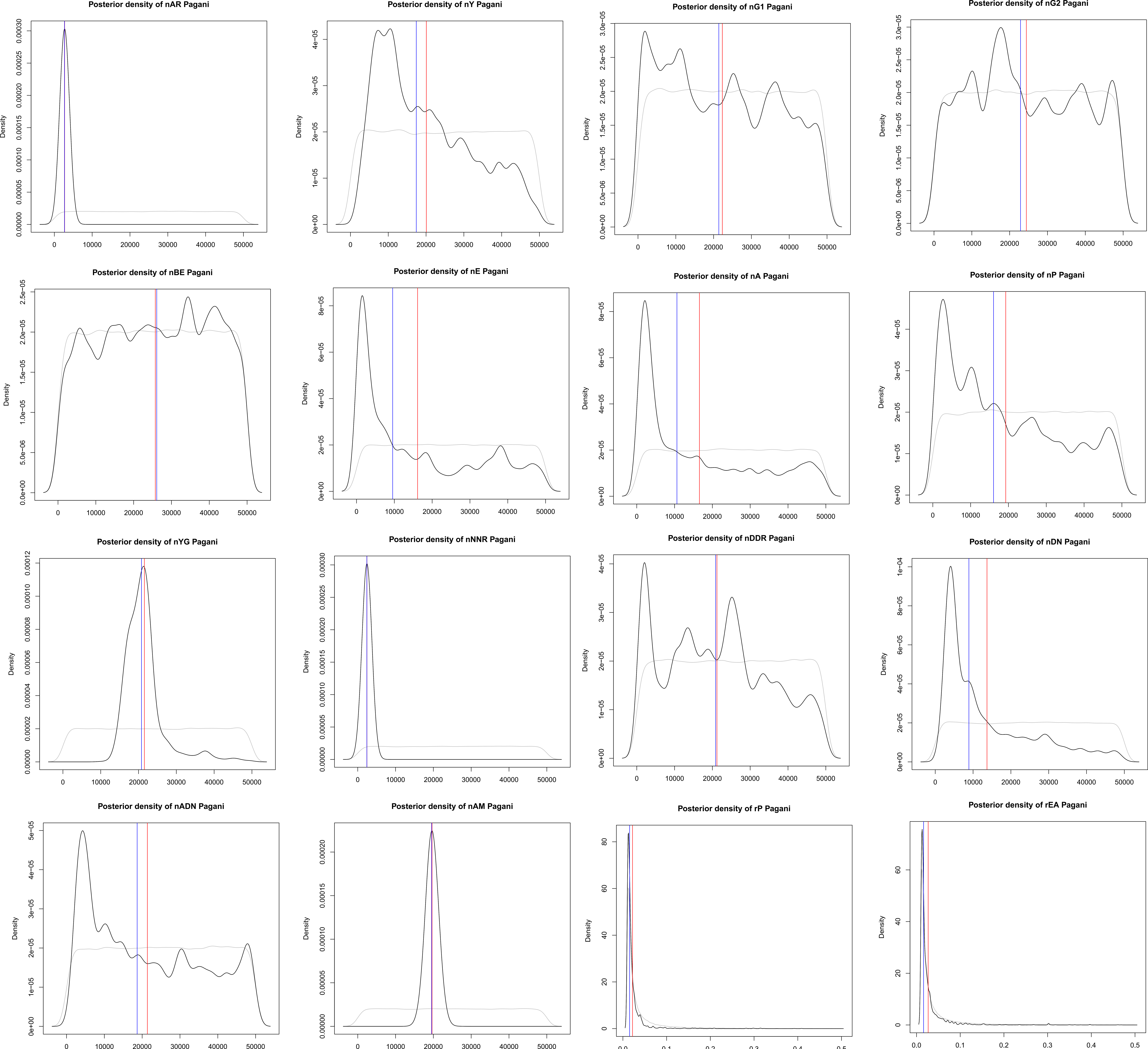
Posterior density of nAR Pagani
Posterior density of nY Pagani
Posterior density of nG1 Pagani
Posterior density of nG2 Pagani
Posterior density of nBE Pagani
Posterior density of nE Pagani
Posterior density of nA Pagani
Posterior density of nP Pagani
Posterior density of nYG Pagani
Posterior density of nNNR Pagani
Posterior density of nDDR Pagani
Posterior density of nDN Pagani
Posterior density of nADN Pagani
Posterior density of nAM Pagani
Posterior density of rP Pagani
Posterior density of rEA Pagani
Density

Figure S7. **Posterior density of the divergence times and the admixture times estimated using the Papuan sample from Pagani et al. (2016)**. The plots have the same features of Figure S2.

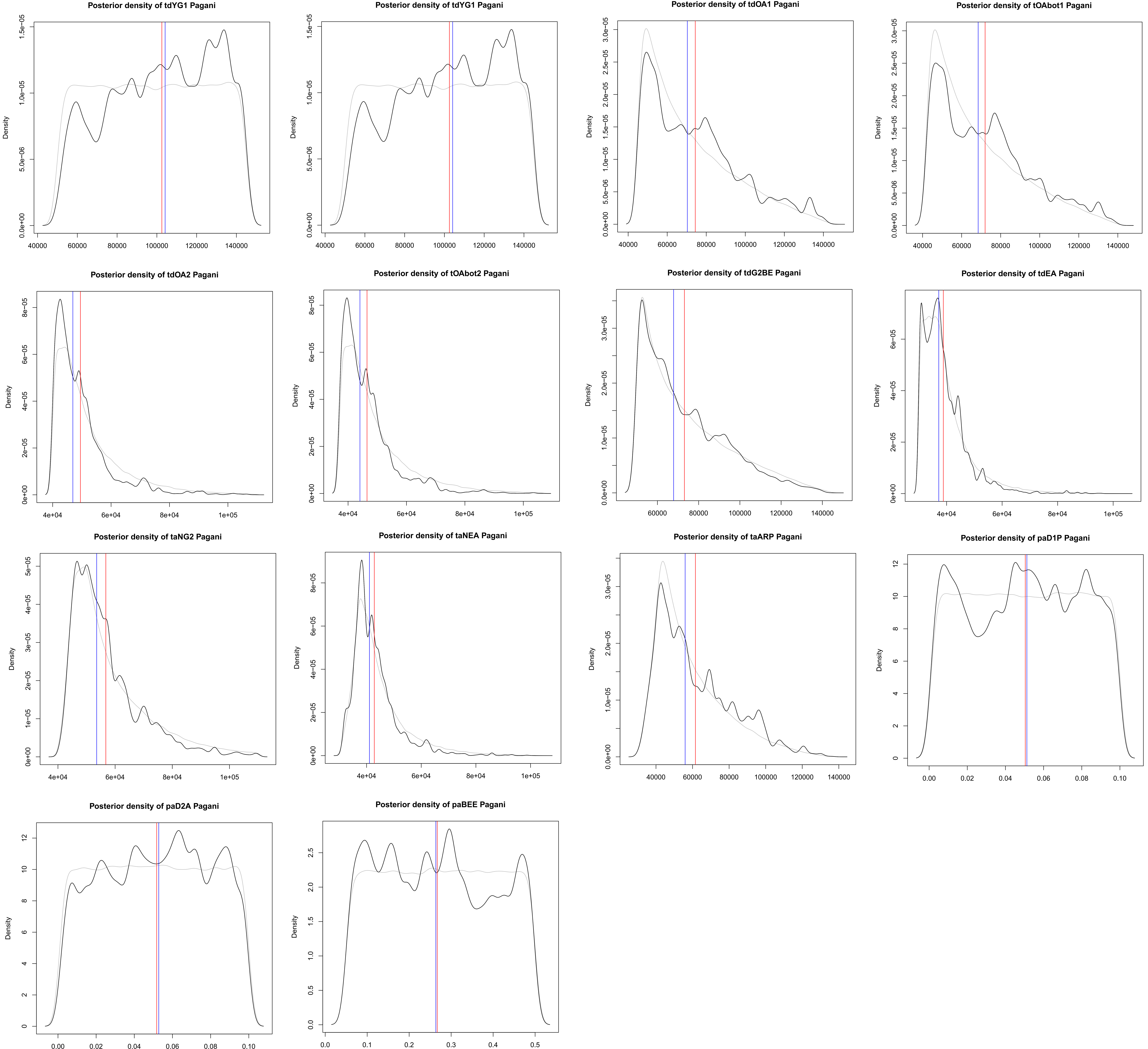
Posterior density of tdYG1 Pagani
Posterior density of tdYG1 Pagani
Posterior density of tdOA1 Pagani
Posterior density of tOAbot1 Pagani
Posterior density of tdOA2 Pagani
Posterior density of tOAbot2 Pagani
Posterior density of tdG2BE Pagani
Posterior density of tdEA Pagani
Posterior density of taNG2 Pagani
Posterior density of taNEA Pagani
Posterior density of taARP Pagani
Posterior density of paD1P Pagani
Posterior density of paD2A Pagani
Posterior density of paBEE Pagani
Density

Figure S8. **Posterior density of the admixture rates estimated using the Papuan sample from Pagani et al. (2016)**. The plots have the same features of Figure S2.

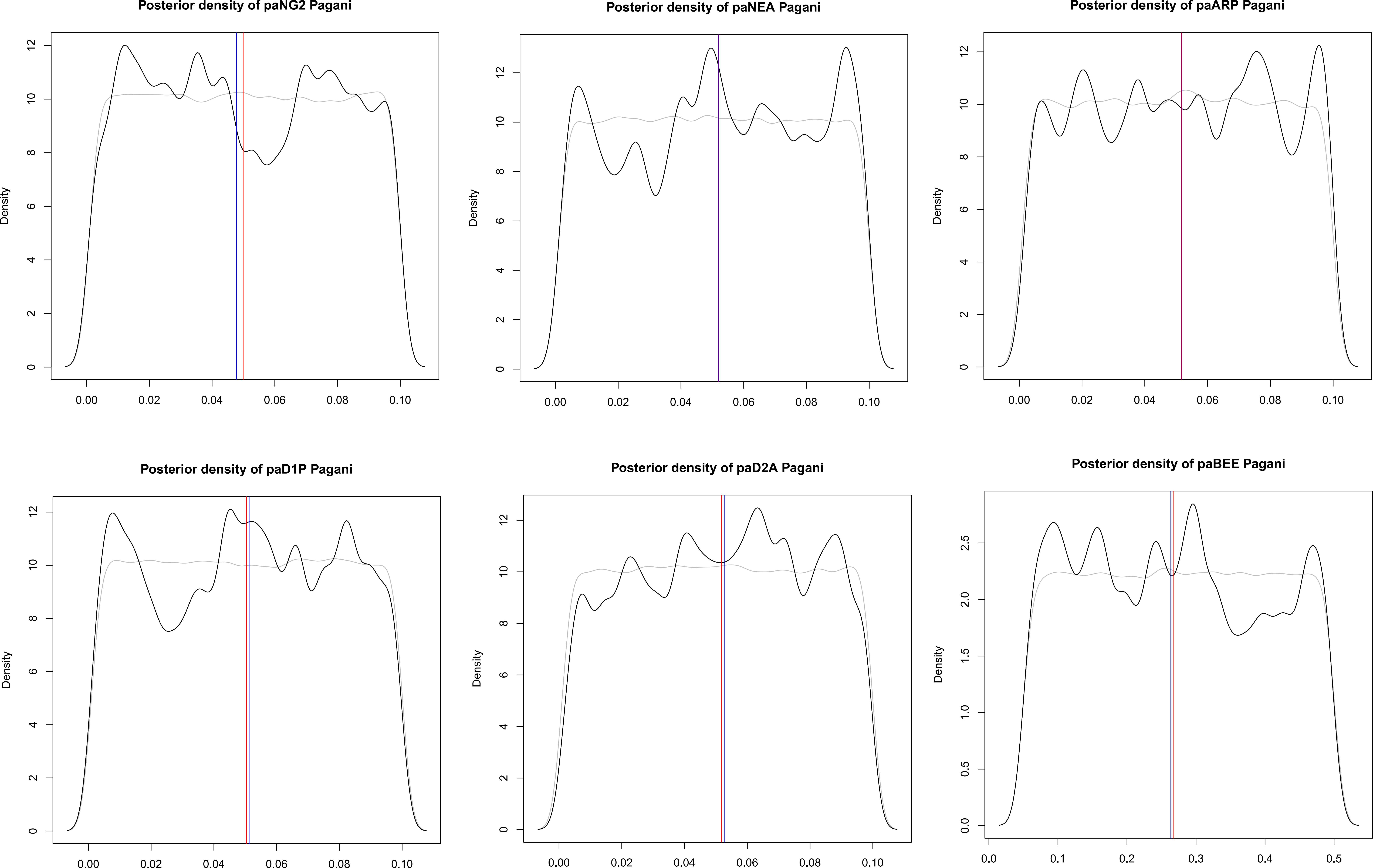

Posterior density of paNG2 Pagani
Posterior density of paNEA Pagani
Posterior density of paARP Pagani
Posterior density of paD1P Pagani
Posterior density of paD2A Pagani
Posterior density of paBEE Pagani
Density

Figure S9. **Posterior density of the migration rates estimated using the Papuan sample from Pagani et al. (2016)**. The plots have the same features of Figure S2.

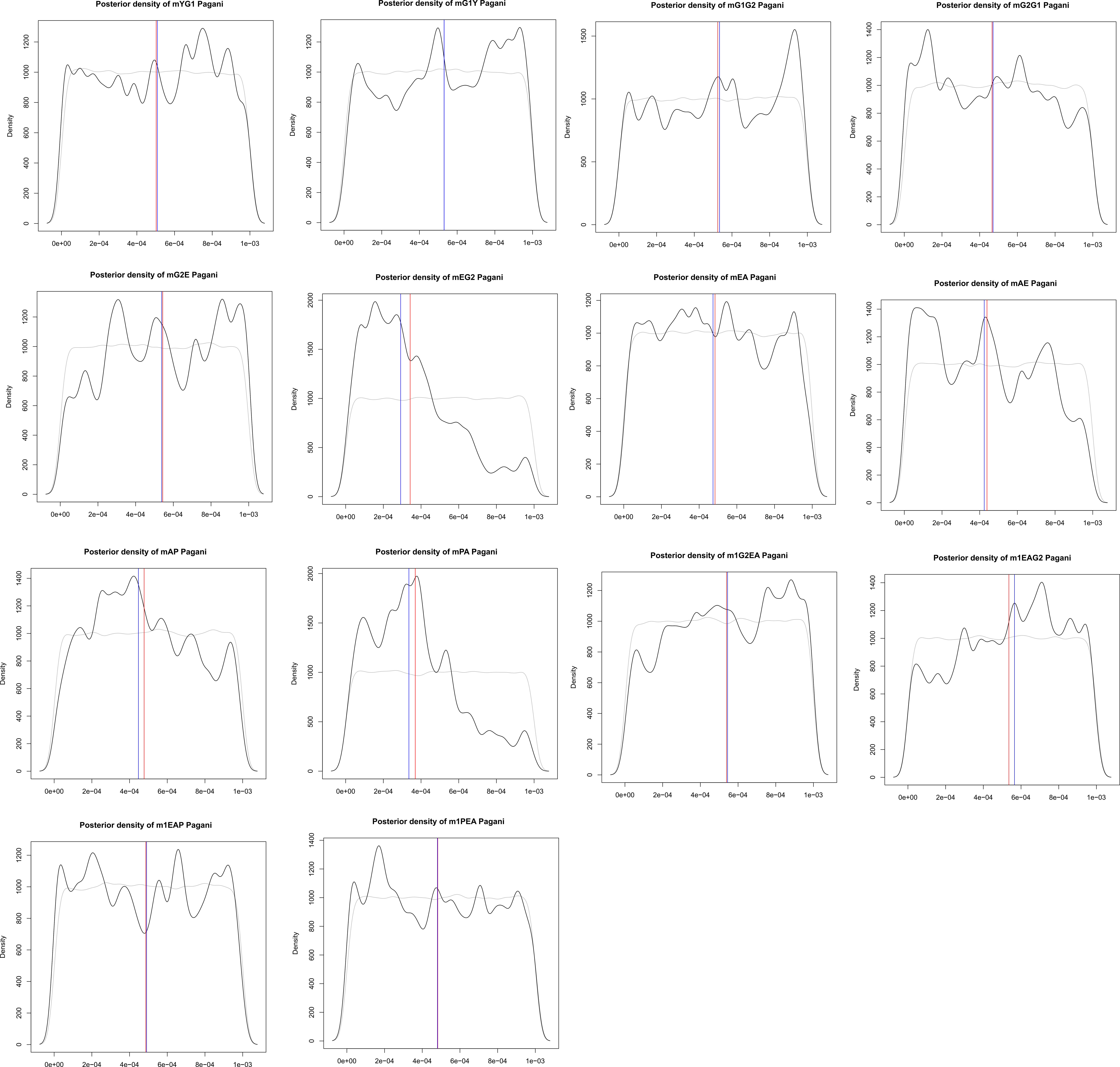

Posterior density of mYG1 Pagani
Posterior density of mG1Y Pagani
Posterior density of mG1G2 Pagani
Posterior density of mG2G1 Pagani
Posterior density of mG2E Pagani
Posterior density of mEG2 Pagani
Posterior density of mEA Pagani
Posterior density of mAE Pagani
Posterior density of mAP Pagani
Posterior density of mPA Pagani
Posterior density of m1G2EA Pagani
Posterior density of m1EAG2 Pagani
Posterior density of m1EAP Pagani
Posterior density of m1PEA Pagani
Density
0e+00
2e-04
4e-04
6e-04
8e-04
1e-03

Figure S10: The model below represents a simplified version of the most supported model (MD) showing the main demographic parameters. To ensure readability migrations and admixture events are not shown.

Multiple Dispersal Model

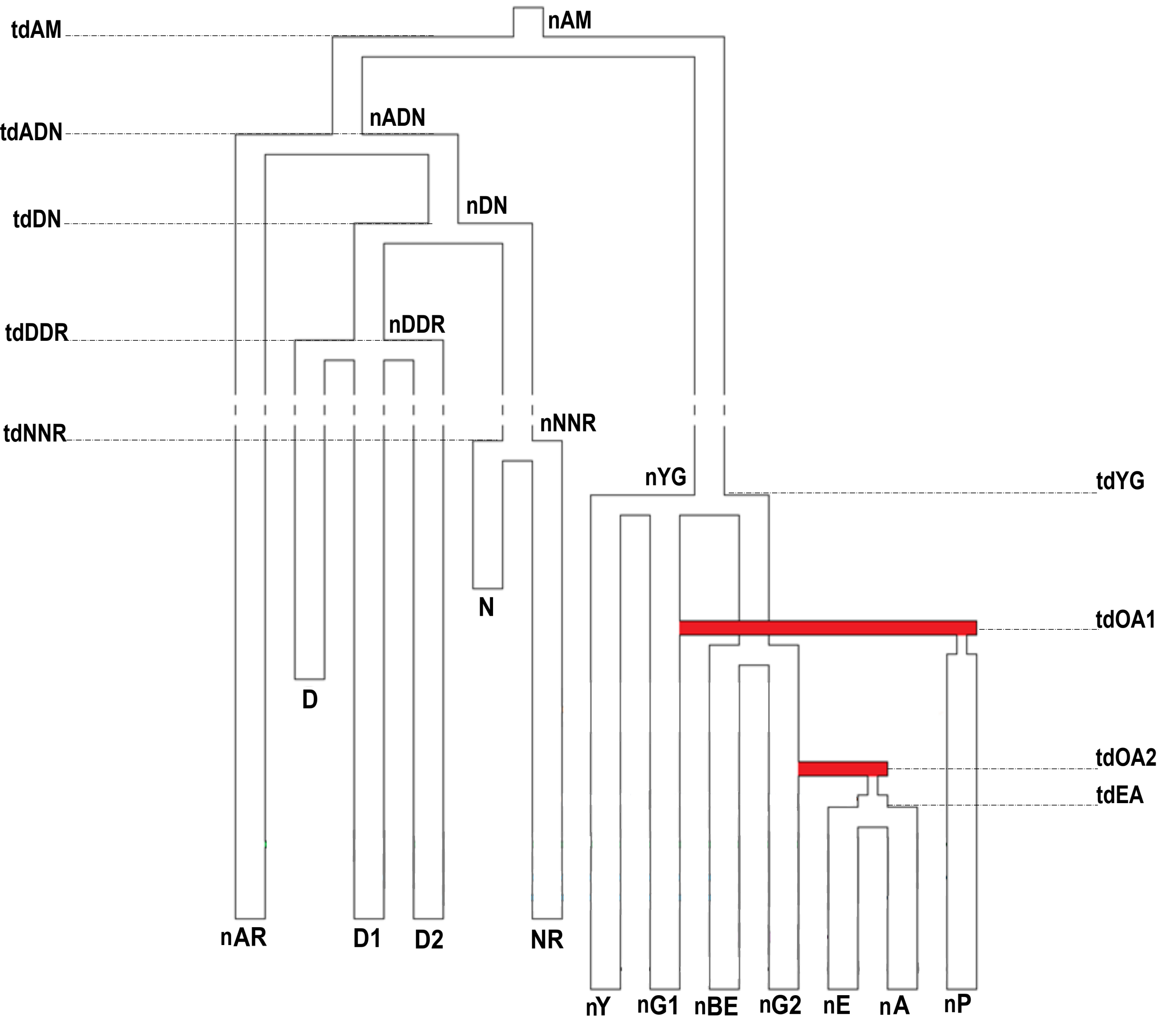

Supplement: Supplementary file 1 [file genes-11-01510-s001.pdf]
